# Application of Novel Triazolium-Containing Hydrogels to Cotton Fabrics: Evaluation of Their Flame Retardancy and Antibacterial Properties

**DOI:** 10.1021/acsomega.5c00281

**Published:** 2025-05-23

**Authors:** Nejmi Söyler, Eylen Sema Dalbaşı, Süleyman Ilhan, Hayati Türkmen

**Affiliations:** † Graduate School of Natural and Applied Sciences, Materials Science and Engineering, 37509 Ege University, Bornova, 35100 Izmir, Turkey; ‡ Department of Textile Engineering, 37509 Ege University, Bornova, 35100 Izmir, Turkey; § Department of Biology, Faculty of Engineering and Natural Sciences, 52953 Manisa Celal Bayar University, Manisa 45140, Türkiye; ∥ Department of Chemistry, Faculty of Science, 37509 Ege University, Bornova, 35100 Izmir, Turkey

## Abstract

A novel series of triazolium ionic salts were synthesized and characterized using Fourier transform infrared spectroscopy as well as ^1^H and ^13^C nuclear magnetic resonance spectroscopy. The thermal degradation kinetics and activation energy of the ionic salts were studied using Kissinger–Akahira–Sunose, Flynn–Wall–Ozawa, and Starink methodologies. The results indicated that the thermal degradation mechanism of the synthesized triazolium flame retardants is influenced by the mono- and dicationic triazolium rings with different alkyl chain lengths. The activation energy increased with the decrease in the alkyl chain length and the addition of a triazolium ring. Triazolium-containing hydrogels were prepared and applied to cotton fabrics to enhance their flame-retardant and antibacterial properties. The vertical flammability test results confirmed that PBDIL12_20, which contained 20 wt % dicationic ionic salt, exhibited the highest flame retardancy among the tested samples. All the cotton fabrics treated with the triazolium hydrogels exhibited excellent antibacterial activity against Staphylococcus aureus and *Escherichia coli*, achieving bacteriostatic rates of >99%. This study presents a novel method for the development of flame-retardant and antibacterial cotton fabrics, which can be used in protective clothing to safeguard skin from fire and health hazards. The triazolium salts on normal cells (the human keratinocyte cell line (HaCaT) and the human dermal fibroblast cell line (BJ)) were used for MTT viability analysis.

## Graphical abstract

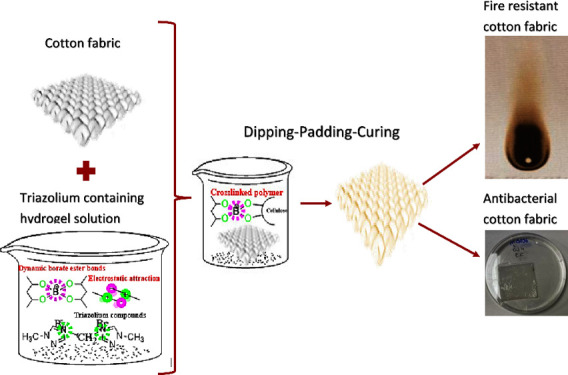

## 1 Introduction

Flammable textile materials, including cotton fabrics, are responsible for 20% of fire-related disasters. Studies focused on enhancing the flame-retardant properties of cotton fabrics have garnered considerable attention.^1,2^ Therefore, the identification of highly cost-effective and environmental-friendly methods for reducing the flammability of cotton fabric materials is crucial for public safety.^3^ Several applications necessitate the integration of advanced smart functionalities into textile fabrics for achieving unique characteristics, such as enhanced thermal stability, flame retardancy and antibacterial protection.^4,5^ The development of multifunctional textiles that possess both flame-retardant and antibacterial properties will help enhance safety by creating barriers against fire and health hazards, improving the protection of individuals and property.^6,7^ The adoption of novel alternative chemicals is gaining increasing interest for developing environmentally friendly textiles, which are commonly referred to as ‘green textiles’ or ‘eco-friendly textiles’.^8^

The flame retardancy of cotton fabrics have been improved using diverse types of materials. For instance, (a) Attia et al. utilized various nanoparticle materials to enhance the thermal stability, flame retardancy and antibacterial properties of textiles. However, the incorporation of nanoparticles into fibers negatively affected the tensile strength of the treated textile fabrics.^9^ (b) Zhang et al. demonstrated that halogenated compounds, including pentabromodiphenyl ether, decabromodiphenyl ether and polychlorinated biphenyls, possess effective flame-retardant properties. However, the use of these compounds has been restricted in several countries because of their hazardous effects on humans and animals, leading to extensive scrutiny and regulatory measures.^10^ (c) Goutham et al. proposed metal hydroxides, such as Al­(OH)_3_ and Mg­(OH)_2_, as alternatives to halogenated flame retardants because of their ability to absorb considerable amounts of heat at high temperatures. However, to achieve effective flame retardancy, high concentrations of metal hydroxides are required, which negatively impacts the mechanical properties of the resulting materials.^11^ (d) Rosace et al. developed several organophosphorus-based flame retardants, including phosphates, phosphoramides, phosphonates and phosphonium salts; however, only a few of these materials were successful.^12^ (e) Yu et al. developed the fire-resistant thermal protective composite aerogel combining aramid nanofibers, polyethylene glycol, Fe_3_O_4_, and polyaniline.^13^ Previous studies have commonly focused on inorganic antibacterial agents, such as Ag and Cu nanoparticles, which possess broad-spectrum and high-efficiency antibacterial properties. However, the potential damage caused by the release of nanoscale metals into the environment is a considerable issue.^14^

In the past decade, ionic salts have emerged as a fast-developing area of chemical research focused on new materials.^15^ Multidisciplinary studies on ionic salts are emerging in fields such as chemistry, material science, chemical engineering and environmental science.^16^ The superior efficiency, higher performance and lower hazard risks of ionic salts enable them to replace conventional organic solvents in numerous processes.^17^ Compared with monocationic ionic salts, dicationic ionic salts have higher thermal and chemical stability, higher solubility of compounds, enhanced surface properties and lower volatility.^18^ 1,2,4-triazole derivatives exhibit significant potential as flame retardants due to their high nitrogen content.^19^ These compounds contribute to the formation of stable, heat-resistant char layers during combustion, thereby enhancing their flame-retardant properties.^20^ Moreover, they demonstrate excellent thermal stability,^21^ maintaining structural integrity at elevated temperatures and resisting decomposition.^22^ In addition to their flame-retardant characteristics, 1,2,4-triazole derivatives possess important biological activities, including antiviral, antibacterial,^23^ and antifungal^24^ effects. Moreover, the alkyl substituents of different lengths and different counteranions around the triazolium compounds can affect their antibacterial and thermal properties.^25^

Triazolium cations are suitable for synthesizing specialized ionic salts for ‘fully organic’ applications. Furthermore, hydrogels are promising flame-retardant materials owing to their ability to prevent water loss and form protective layers.^26^ When exposed to fire, water in the hydrogels gradually evaporates, absorbing heat and delaying the combustion process.^1,27^ Therefore, the combination of hydrogels with cotton fabrics can reduce the flammability of the resulting textile materials when exposed to fire. Additionally, the hydrogels containing various antibiotics and antibacterial agents have the ability to kill bacteria and prevent infections when used in textile materials.^28^

This study aimed to synthesize novel triazolium salts, both dicationic and monocationic salts, for preparing triazolium-based ‘green’ hydrogels. Furthermore, we evaluated the flame-retardant and antibacterial properties of the novel triazolium-containing hydrogels applied to cotton fabrics. Thermal stability is a critical factor that influences their flame-retardant treatment. Understanding the thermal degradation behavior is important for analyzing the flame-retardant properties and charcoal mechanism of the synthesized materials.^29^ The Kissinger–Akahira–Sunose (K–A–S), Flynn–Wall–Ozawa (F–W–O) and Starink kinetic methods were applied to study the activation energy of the synthesized flame retardants. Furthermore, this paper focuses on the thermal degradation kinetics of triazolium ionic salts and discusses the effects of activation energy on the triazolium hydrogel-treated cotton fabrics for achieving efficient flame retardancy. This study develops multifunctional cotton fabrics by applying triazolium-containing hydrogels, broadening their potential applications, such as in the production of clothing, home textiles, and industrial products.

## 2 Material and Method

### 2.1 Materials

Pretreated cotton fabric with a density of 220 g/m^2^ was sourced from the local market in Izmir. The details of the chemicals used in the present study, including their molecular structure, molecular weight and CAS number, are provided in the Supporting Information (SI; Table S1). 1,2-Dibromoethane, 1,5-dibromopentane and 1-bromopentane were purchased from Sigma-Aldrich and used without any additional purification. 1H-1,2,4-triazole was purchased from AFG Bioscience. Poly­(vinyl alcohol) (PVA; degree of hydrolysis is approximately 90 mol %) was purchased from Blab Co., Ltd. Other chemicals and all the solvents employed in this study were obtained from BRK Chem. and used as received.

### 2.2 Synthesis of New Triazolium Salts

The new triazolium salts was synthesized in two steps. In the first step, 1H-1,2,4-triazole (1.5 g) was added to 15 mL of tetrahydrofuran (THF) solution comprising K_2_CO_3_ (4.5 g) and stirred at 500 rpm for 60 min. Thereafter, CH_3_I (1.5 mL) was added dropwise over a period of 20–35 min under an argon gas flow at 0 °C in an ice bath. The reaction mixture was stirred at 25 °C for 1 day. THF was removed via evaporation under vacuum conditions, and dichloromethane was added. Subsequently, the mixture was filtered to remove solids. Finally, dichloromethane was evaporated to yield 1-methyl-1,2,4-triazole as a light-yellow liquid (Figure 1). The nuclear magnetic resonance (NMR) spectra were recorded in CDCl_3_ and matched with the literature reports.

**Figure 1:**
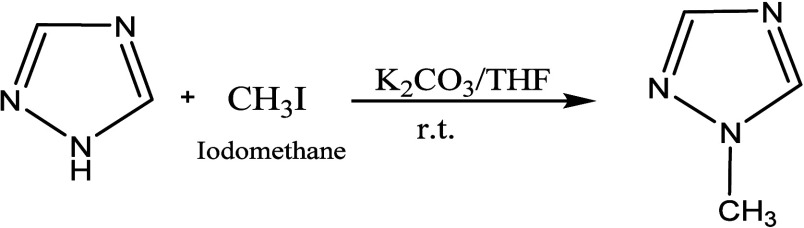
Synthesis of 1-methyl-1,2,4 triazole.

In the second step, 1-methyl-1,2,4-triazole and each bromoalkane compound were refluxed at 110 °C for 24–30 h. After cooling, the resulting sticky solid was washed three times with dichloromethane and once with diethyl ether. Thereafter, the solvent was completely removed under vacuum to yield an amorphous, hygroscopic and white powder (Figure 2). The NMR spectra were recorded in D_2_O. The synthesized mono- and dicationic triazolium ionic salts were labeled IL15, DIL15 and DIL12.

**Figure 2:**
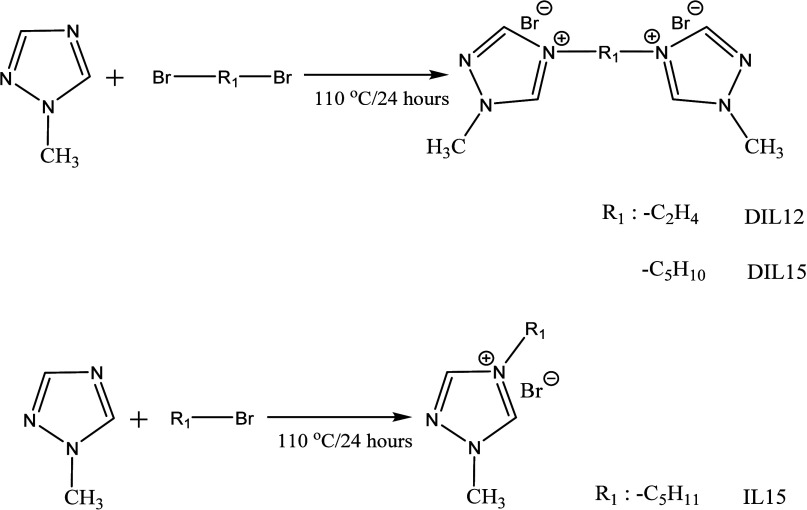
Synthesis of mono- and dicationic triazolium ionic salts.

### 2.3 Preparation of Triazolium-Containing Hydrogels

The new triazolium-containing hydrogels were synthesized using the following procedure. PVA solid (1.5 wt %) was added to deionized water and completely dissolved using a magnetic stirrer at 95 °C for 2 h. Thereafter, a borax solution was prepared by dissolving borax (2 wt %) in deionized water. Subsequently, the two aforementioned solutions were combined under stirring, resulting in the immediate formation of a hydrogel. The hydrogel formed using PVA and borax was designated as PB. The hydrogels formed using PVA, borax and triazolium salts were labeled as PBIL15, PBDIL15 and PBDIL12. The triazolium contents in the as-prepared hydrogels were 10, 20, and 30 wt %, and the synthesized samples were referred to as PBIL15_10, PBDIL15_10, PBDIL12_10, PBDIL12_20 and PBDIL12_30.

### 2.4 Preparation of Triazolium Hydrogel-Treated Cotton Fabrics

A pretreated cotton fabric with the dimensions of 22 × 17 × 0.1 cm^3^ was placed into a glass mold and allowed to absorb the triazolium-containing hydrogel mixture, resulting in a highly efficient hydrogel-treated cotton fabric. The treated cotton fabrics were passed through a laboratory padder to obtain a wet pick-up of 95% at a speed of 2 m/mins and pressure of 3 kg/cm^2^. Afterward, the fabrics were cured at 130 °C for 3 min. The amount of material deposited on the cotton fabrics was determined by measuring the mass difference before and after the treatment using eq 1.

$$A=(W_{f}-W_{i})/W_{i}\times 100$$ 1

where *A* represents the percentage mass increase, and *W*_i_ and *W*_f_ are the masses of the fabric sample before and after treatment, respectively. Figure 3 depicts the schematic of the preparation process and formation mechanism of the new triazolium-containing hydrogels. During the cross-linking process, when borax dissolves in water, it transforms into tetrahydroxy borate ions and boric acid via the following chemical reactions:

$${\mathrm{Na}}_{2}B_{4}O_{7}+7H_{2}O\to 2B(\mathrm{OH}{)}_{3}+2B(\mathrm{OH}{{)}^{-}}_{4}+2{\mathrm{Na}}^{+}$$

$$B(\mathrm{OH}{)}_{3}+2H_{2}O⇌B(\mathrm{OH}{{)}^{-}}_{4}+H_{3}O^{+}$$

**Figure 3:**
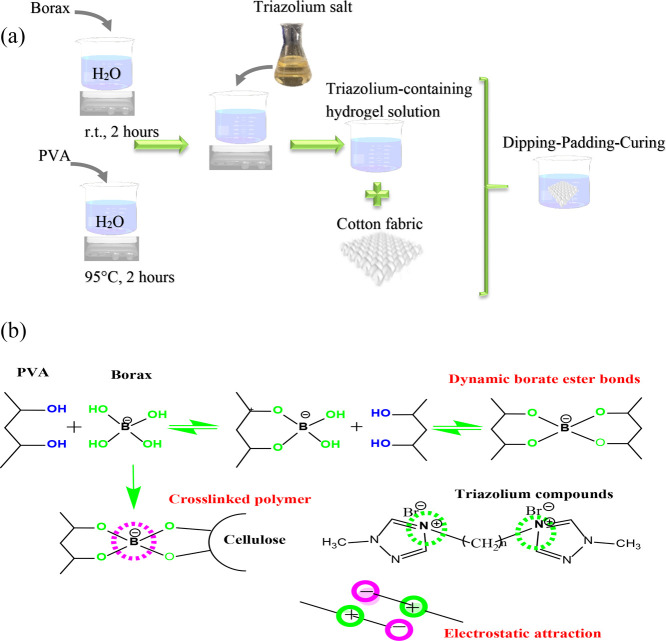
Schematic diagram of the preparation process (a) and formation mechanism of new triazolium-containing hydrogels (b).

Thereafter, the hydroxyl groups of PVA form a complex with tetrahydroxy borate ions.^27,30^ Moreover, the interactions involving dynamic boronate ester bonds within the complex were reversible, endowing the resulting hydrogels with flame-retardant and antibacterial properties. In addition to the microcrystalline domain cross-linking points, tetrahydroxy borate ions can form hydrogen bonds with cellulose along the polymeric chains. Furthermore, electrostatic attractions may exist between the positively charged groups of the triazolium salt ions and the negatively charged borate ions.^31^ Therefore, the interactions between the prepared hydrogel system and cotton fabric are anticipated to confer the as-prepared hydrogel-containing cotton fabrics with highly effective flame-retardant and antibacterial properties.

### 2.5 Characterization

The structures of the synthesized triazolium salts (DIL12, DIL15 and IL15) were confirmed via ^1^H- and ^13^C NMR analyses. The chemical structures of DIL12, DIL15, IL15, PB, PBDIL12_10, PBDIL15_10 and PBIL15_10 were characterized using Fourier transform infrared (FTIR) spectroscopy. Attenuated total reflection (ATR)-FTIR spectroscopy was conducted using a Nicolet IS50 FTIR spectrometer to analyze the chemical structures of PBIL15_10, PBDIL15_10, PBDIL12_10, PBDIL12_20 and PBDIL12_30. The FTIR analysis was performed over a spectral range of 4000–400 cm^–1^ with a resolution of 2 cm^–1^. The surface morphology of the treated cotton fabrics was observed using a Quanta 250 FEG scanning electron microscope^4^ at a voltage of 20 kV. All samples were coated with a thin layer of gold prior to observation. The contents of bromine (Br), carbon (C), oxygen (O), and nitrogen (N) in the treated cotton fabrics were analyzed using Quantax energy-dispersive spectroscopy (EDS).

### 2.6 Thermokinetic Analysis

The thermogravimetric analysis (TGA) of the synthesized triazolium salts (DIL12, DIL15 and IL15) was performed using a Shimadzu TG-50 instrument (Japan). The samples were heated at the rates of 10, 20, 30, and 40 °C/mins with a nitrogen flow rate of 10 mL/mins over a temperature range of 0–800 °C. TGA was performed using approximately 10.0 mg of each sample. To evaluate the decomposition kinetics of the triazolium salts, three methodologies were employed, as presented in Table 1. These iso-conversional models enabled the determination of kinetic parameters using the nonisothermal TGA data obtained at different heating rates (β). The weight loss observed during TGA was used to calculate the mass fraction of conversion (α) using eq 2, where *m*_o_, *m*_t_ and *m*_f_ represent the initial, instantaneous and final mass of the sample, respectively. The activation energy (*E*_a_) was derived from the slope across a broad range of conversion values.^32^

$$\propto =\frac{m_{o}-m_{t}}{m_{o}-m_{f}}$$ 2

**Table 1:** Used Kinetic Methods for Calculation of Activation Energy in the Study

isoconversional methods	expressions	plots	references
K–A–S	ln(β /*T* ^2^) = ln(*AR*/*E*_a_) – *E*_a_/*RT*	ln(β /*T* ^2^) versus 1000/*T*	^33^
F–W–O	ln β = ln[*AE*_a_/*RF*(α)] – 2.315 – 0.4567*E*_a_/*RT*	ln β versus 1000/*T*	^34^
Starink	ln β/*T* ^1.92^ = ln(*AR*/*E*_a_) – 1.0008(*E*_α_ *RT*)	ln(β/*T* ^1.92^) versus 1/*T*	^35^

### 2.7 Equilibrium Swelling Ratio and Water Retention Behavior

The water retention capacity of the hydrogels was assessed by determining their equilibrium swelling ratio in distilled water. Three hydrogel samples were immersed in distilled water at room temperature until they reached an equilibrium state. Once the equilibrium was achieved, the samples were taken out and weighed, recording their swollen weight as *W*_e_. Subsequently, the hydrogels were dried in an oven at 90 °C until a constant weight was achieved. After drying, the hydrogels were weighed, and their dry weight was noted as *W*_o_ *.* The equilibrium swelling ratio was subsequently calculated using eq 3.^1^ Here, *W*_o_ refers to the weight of the fully dried hydrogel after heating at 90 °C, and *W*_e_ represents the weight of the hydrogel when it reached the equilibrium swelling state.

$$\mathrm{ESR}(\%)=\frac{W_{e}-W_{o}}{W_{o}}$$ 3

For the application of the hydrogel-treated cotton fabrics as flame-retardant materials in fire-protective clothing, their water retention capacity should be evaluated under standard atmospheric conditions. For this purpose, the fabric samples were weighed at hourly intervals until a constant weight was achieved. Thereafter, the water retention ratio of the hydrogels was determined using eq 4,^26^ where *W*_i_ and *W*_t_ denote the weights of the initial and dried hydrogels, respectively.

$$\mathrm{Water}\;\mathrm{retention}\;\mathrm{ratio}(\%)=1-\frac{W_{i}-W_{t}}{W_{i}}\times 100\%$$ 4

### 2.8 Vertical Flammability and Limiting Oxygen Index (LOI) Tests

Vertical flammability tests were conducted in accordance with BS 5438-1989, utilizing an SDL ATLAS M233B model automatic vertical flammability cabinet (Textile and Clothing Research and Application Center, Turkey). Strips of the pretreated and flame-retardant cotton fabric samples were cut into segments with the dimensions of 220 × 170 mm. Afterward, they were vertically ignited from the bottom for 10 s using a propane burner, with the flame length set to 40 ± 2 mm. The flame-retardant properties were assessed by measuring the char length of the samples after the burning period of 10 s. Furthermore, a JF-5 oxygen index gauge was used for the Limiting Oxygen Index (LOI) test which was used to determine the minimum amount of oxygen required to sustain the flaming combustion of the treated fabrics. Based on ASTM D 2863-77, the test samples were processed into segments with the dimensions of 140 × 52 mm.

### 2.9 TGA-FTIR Analysis of the Treated Cotton Fabrics

Thermogravimetric analysis coupled with Fourier Transform Infrared Spectrometry was performed using a Hitachi STA 7300 thermogravimetric analyzer and a Shimadzu IRAffinity-1 FTIR spectrometer. Treated cotton fabric samples (10 ± 0.5 mg) were heated from 25 to 760 °C at a rate of 20 °C/mins under a nitrogen flow rate of 60 mL/mins. The resulting volatile decomposition products were transferred via a heated stainless steel line to the FTIR gas cell. The gas cell and insulating pipe were maintained at 300 °C in order to prevent condensation and secondary reactions. The gas components were then recorded as the absorption peaks in the 4000–600 cm^–1^ region.

### 2.10 Antibacterial Testing of the Hydrogels Applied to Cotton Fabrics

The antibacterial activity of the triazolium-containing hydrogel-treated cotton fabrics was assessed using Escherichia coli (E. coli) and Staphylococcus aureus (S. aureus). The antibacterial activity of the cotton fabrics was assessed using the AATCC 100 standard methodology.

### 2.11 Mechanical Properties of the Treated Cotton Fabrics

The mechanical performance of the treated textiles was evaluated using a strength tester. For the experiment, the samples were cut into pieces with the dimensions of 200 × 50 mm. The testing was conducted using a holding distance of 100 mm and stretching velocity of 100 mm/mins. Three measurements were conducted for each type of the treated cotton fabrics and the pretreated cotton sample.

### 2.12 Cell Culture and MTT Assay

To evaluate the effects of the synthesized triazolium salts on normal cells, the human keratinocyte cell line (HaCaT) and the human dermal fibroblast cell line (BJ) were used for MTT viability analysis. Both cell lines were cultured in Dulbecco’s Modified Eagle Medium (DMEM) supplemented with 10% fetal bovine serum (FBS), 1% l-glutamine, and 1% penicillin-streptomycin. Cells were maintained under standard conditions in a humidified incubator with 5% CO_2_ at 37 °C and were used in the logarithmic growth phase for all experiments.

For the MTT assay, cells were seeded into 96-well plates at a density of 5 × 10^3^ cells per well and allowed to adhere for 24 h. After stabilization, the cells were treated with various concentrations (1, 10, 100, 250, and 500 μM) of the triazolium salts and incubated for 48 h. The final concentration of DMSO used to dissolve the compounds did not exceed 0.1% in any well, and the same concentration of DMSO was used in the vehicle control group. At the end of each incubation period, 20 μL of MTT solution (5 mg/mL in PBS) was added to each well, and the plates were incubated for an additional 4 h at 37 °C. Following incubation, the medium was removed, and 200 μL of DMSO was added to each well to solubilize the formazan crystals. The plates were gently agitated for 10 min at room temperature, and absorbance was measured at 570 nm using a microplate reader (Tecan). All experimental conditions were tested in triplicate. Cell viability was calculated as the percentage of absorbance values relative to the untreated control group.

## 3 Results and Discussion

### 3.1 Characterization of Triazolium Salts

The ^1^H and ^13^C NMR spectral data of 1-methyl-1,2,4-triazole, DIL12, DIL15 and IL15 are presented in Table 2, and the analysis results are provided in the Supporting Information (SI; Figures S1–S8). The FTIR spectra and the assigned functional groups of the triazolium salts (DIL12, DIL15 and IL15) are presented in Figure 4 and Table 3, respectively. The IR spectra of DIL12 and DIL15 broader and more complex because of the presence of two charged centers, which leads to a higher degree of ionic interactions and hydrogen bonding. Furthermore, the longer alkyl chain in DIL15 enhances its flexibility and vibrational complexity, particularly in the C–H and O–H regions. In contrast, IL15 exhibits a simpler IR spectrum with sharper peaks, as it has a less complex structure and fewer interactions between the cation and counterion. The absence of a second triazolium unit and the presence of a single alkyl chain simplify the vibrational modes. Additionally, the IL15 spectrum demonstrates a less intense and narrower peak in the O–H stretching region compared with the DIL12 and DIL15 spectra. Because IL15 is monocationic, it plausibly has fewer interactions with moisture and less extensive hydrogen bonding than the dicationic salts, resulting in the narrower O–H stretch. Furthermore, the difference in the IR spectra of these salts can be attributed to the variations in their structural composition.

**Table 2:** ^1^H NMR and ^13^C NMR Spectra Triazolium Salts

sample	spectra results
1-methyl-1,2,4-triazole	appearance: light yellow liquid
	yield: 65%
	^1^H NMR (400 MHz, CDCl_3_, 25 °C): δ 7.99 (s, 1H), 7.85 (s, 1H), 3.87 (s, 3H)
	^13^C NMR (100 MHz, 25 °C): δ 151.90, 143.47, 36.04
DIL12	appearance: white solid
	yield: 95%
	melting point: 294 °C
	^1^H NMR (400 MHz, D_2_O, 25 °C): δ 9.86 (s, 2H), 8.88 (s, 2H), 4.87 (s, 4H), 4.07 (s, 6H)
	^13^C NMR (100 MHz, 25 °C): δ 144.42, 46.34, 39.03
DIL15	appearance: white solid
	yield: 80%
	melting point: 175 °C
	^1^H NMR (400 MHz, D_2_O, 25 °C): δ 9.71 (s, 2H), 8.79 (s, 2H), 4.26 (t, 4H), 4.05 (s, 6H), 1.92 (t, 4H), 1.40 (s, 2H)
	^13^C NMR (100 MHz, 25 °C): δ 144.37, 142.30, 47.89, 38.78, 28.40, 22.24
IL15	appearance: white solid
	yield: 84%
	melting point: 120 °C
	^1^H NMR (400 MHz, D_2_O, 25 °C): δ 9.65 (s, 1H), 8.74 (s, 1H), 4.20 (t, 2H), 4.01 (s, 3H), 1.81 (t, 2H), 1.23 (s, 4H), 0.77 (t, 3H)
	^13^C NMR (100 MHz, 25 °C): δ 150.52, 144.37, 48.28, 37.86, 28.55, 27.43, 21.31, 13.06

**Figure 4:**
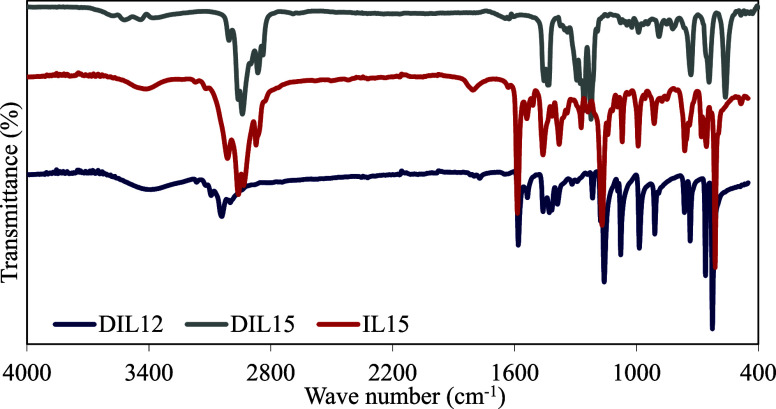
FT-IR spectrum image of DIL12, DIL15, and IL5 triazolium salts.

**Table 3:** FT-IR Spectral Characteristics Functional Group Assignment of Triazolium Salts

	wavenumber range (cm^–1^)			
functional group/assignment	DIL12	DIL15	IL15	literature
O–H stretching (moisture, H-bonding)	3417	3437	3424	^36^
C–H stretching (alkyl chains)	2960	2935	2955	^37^
C=N and C–N stretching (triazolium ring)	1582	1455	1577	^38^
C–H bending (alkyl chains)	986	989	991	^39^

### 3.2 Thermokinetic Analysis of Triazolium Salts

The decomposition profile and associated data of each triazolium salt were gathered through TGA conducted at the heating rates of 10, 20, 30, and 40 °C/mins. The onset decomposition temperature (*T*_onset_), final decomposition temperature (*T*_final_), decomposition percentage (%) and maximum decomposition temperature (*T*_d_) are presented in Table 4. The *T*_onset_ values indicate that the thermal degradation did not occur, as evidenced by the absence of mass loss at temperatures lower than 217 °C. *T*_final_ signifies the temperature at which the complete mass loss is achieved. *T*_*d*_, ranging from 0 to 800 °C for the analyzed samples, corresponds to the peak temperature observed in the TGA thermogram comprising the plot of derivative weight (%) versus temperature (*T*) (Figure 5). The decomposition kinetics were studied to assess the effects of structural factors, such as the presence of an additional cationic head and the length of alkyl chains, on the thermal degradation behavior of triazolium ionic salts.

**Table 4:** Decomposition Temperatures of DIL12, DIL15, and IL5 Samples at 20°C/min Heating Rate

sample	*T*_onset_(°C)	*T*_final_(°C)	*T*_d_(°C)
DIL12	288	420	312
DIL15	234	378	267
IL5	217	291	261

**Figure 5:**
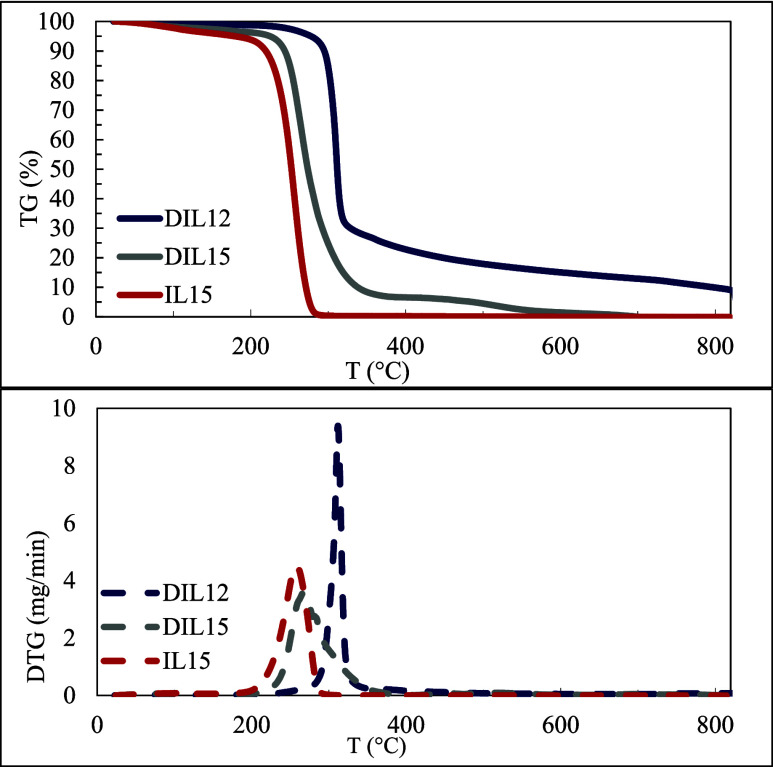
TG and DTG graphs of DIL12, DIL15, and IL5 at 20 °C/mins heating rate.

Kinetic parameters were derived from the nonisothermal thermogravimetric data obtained at different heating rates using the K–A–S, F–W–O and Starink methodologies. In these methodologies, the slope of the linear segments corresponding to each α value yields the activation energy for the decomposition process at each α value. The activation energies of DIL12, DIL15 and IL15 remained consistent across all the three kinetic models. The kinetic parameters derived from these methodologies are presented in SI (Figures S9–S11). Based on Table 5, the decomposition of triazolium ionic salts proceeds in multiple stages, exhibiting different activation energies depending on temperature. This result indicates that additional and simultaneous bond cleavage events may occur, which can explain the fluctuations in the activation energy and ln *A* values with increasing α.^33,40^ The highest average activation energies were in the order of DIL12, DIL15 and IL15. This variation can be attributed to two main factors: (i) the different decomposition mechanisms of the alkyl chains of mono- and dicationic ionic salts and (ii) the breakdown of an additional triazolium ring present in dicationic salts compared with the case of their monocationic counterparts. Moreover, similar behaviors were observed for monocationic and dicationic ionic salts with different alkyl chain lengths (*n* = 2 and 5).

**Table 5:** Calculated Activation Energy (*E*_a_) and Regression (*R* ^2^) Values for DIL 12, DIL 15, and IL 5 Triazolium Salts

		KAS		FWO		STARINK	
sample	α	*R* ^2^	*E*_a_ (kJ mol^–1^)	*R* ^2^	*E*_a_ (kJ mol^–1^)	*R* ^2^	*E*_a_ (kJ mol^–1^)
DIL12	0.1	0.9621	258.341	0.9647	254.662	0.9622	258.517
	0.2	0.9853	236.725	0.9864	234.201	0.9854	236.917
	0.3	0.9924	227.296	0.993	225.283	0.9924	227.497
	0.4	0.9979	223.056	0.9981	221.298	0.9979	223.252
	0.5	0.9936	219.107	0.9942	217.614	0.9936	219.322
	0.6	0.9924	258.798	0.993	255.413	0.9925	258.982
	0.7	0.9954	254.367	0.9957	251.254	0.9954	254.554
	0.8	0.9584	260.536	0.9614	257.453	0.9985	260.735
	0.9	0.9652	287.955	0.9676	283.756	0.9653	288.141
average			**247.354**		**244.548**		**247.546**
DIL15	0.1	0.9866	161.574	0.9971	163.149	0.9966	170.334
	0.2	0.997	149.943	0.9998	175.957	0.9993	164.652
	0.3	0.9995	188.304	0.9928	176.558	0.9992	159.975
	0.4	0.9909	200.808	0.9984	184.369	0.9961	189.05
	0.5	0.9738	216.206	0.9991	173.901	0.9996	211.688
	0.6	0.9945	228.236	0.9974	205.486	0.9979	225.196
	0.7	0.9902	195.737	0.9998	220.626	0.9951	181.516
	0.8	0.9972	176.905	0.9998	220.626	0.997	174.022
	0.9	0.9982	249.062	0.9996	214.349	0.9885	232.182
average			**196.308**		**192.78**		**189.846**
IL15	0.1	0.9997	88.9385	0.9997	92.1608	0.9997	88.9385
	0.2	0.9998	80.0247	0.9998	83.8515	0.9998	80.0247
	0.3	0.9999	100.818	0.9999	103.775	0.9999	100.818
	0.4	0.9969	109.192	0.9973	111.594	0.9969	109.192
	0.5	0.9995	113.562	0.9995	116.061	0.9995	113.562
	0.6	0.9995	113.628	0.9996	116.187	0.9995	113.628
	0.7	0.9999	106.625	0.9999	109.586	0.9999	106.625
	0.8	0.9998	109.258	0.9999	104.012	0.9998	109.258
	0.9	0.9999	100.594	0.9999	112.155	0.9999	100.594
average			**102.516**		**105.487**		**102.516**

### 3.3 Characterization of Triazolium-Based Hydrogels

The FTIR spectra of PB, PBDIL12_10, PBDIL15_10 and PBIL15_10 were analyzed to examine the interactions among the hydrogel components. The FTIR spectra of the hydrogels are presented in Figure 6. An O–H stretching band was observed at approximately 3400 cm^–1^ in all the sample spectra, indicating the presence of hydrogen bonding or retained moisture within the hydrogels.^41^ The C–H stretching bands located at 2920–2930 and 2850–2860 cm^–1^ are consistent across all the spectra, confirming the presence of alkyl chains originating from both the polymer and triazolium salts.^42^ Furthermore, the characteristic C=N and C–N stretching vibration bands at approximately 1590 cm^–1^ reflect the presence of the triazolium ring, emphasizing the effective integration of ionic salts into the polymer matrix.^43^ These observations indicate the successful blending of triazolium-based ionic salts (DIL12, DIL15 and IL15) with PB, as indicated by the shifts in the characteristic peaks.

**Figure 6:**
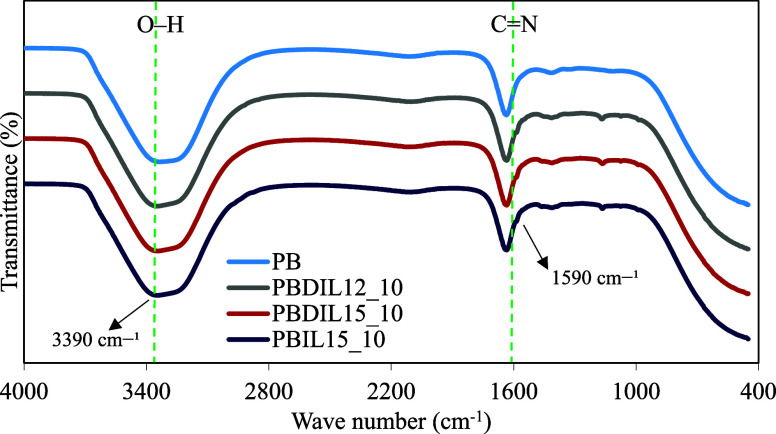
FT-IR spectrum of hydrogels.

TG curves can indicate the thermal stability of flame-retardant hydrogels exposed to heat in an air environment.^26^ As illustrated in SI (Figure S12), the thermal degradation of the synthesized hydrogels occurs in three stages. The onset of degradation occurred between 50 and 200 °C, and the weight loss continued until 250 °C. This initial stage of weight loss was primarily attributed to the evaporation of water and loss of bound water. The second stage that occurred between 250 and 450 °C was associated with the decomposition of alkyl chains and loss of–OH groups in the hydrogel samples. In the third stage, which spanned from 450 to 600 °C, minimal weight loss was observed, indicating that decomposition was nearly complete. Among the samples, the PBDIL12_10 hydrogel exhibited the largest residual weight (10.4%), suggesting that only char residue remained at this time. Moreover, when applied as flame-retardant materials, the synthesized hydrogels can considerably extend the thermal degradation time of untreated cotton fabrics.

### 3.4 Evaluation of the Water Retention Behavior of Hydrogels

The fire-extinguishing mechanism of the hydrogels applied to the cotton fabrics is based on the water absorbed within the hydrogels. A hydrogel with a high water content can effectively extinguish flames, as the absorbed water plays a vital role in determining the flame-retardant properties of the fabrics by cooling and suppressing the flames. The water absorption capacities of the hydrogels can be evaluated by measuring their swelling ratios. The water absorption capacities of the PBDIL12_10, PBDIL15_10 and PBIL15_10 hydrogels in distilled water at 25 °C are presented in Figure 7. The water absorption capacities of >60 g/g indicate that the triazolium ionic salt–containing hydrogels exhibit a strong swelling behavior. The observed variations in the swelling ratio can be attributed to the difference in the hydrophilicity of the synthesized triazolium salts within the hydrogels, which may result in an increase in the average number of water molecules in the system.^26,31^ Figure 8 illustrates that in the case of triazolium-containing hydrogels, > 80% of water was lost after their exposure to air for 96 h, indicating their relatively poor water retention capacity. To ensure that the hydrogel-treated cotton fabrics retain their flame-retardant properties over an extended period at room temperature, further enhancement of the water retention capacity of the hydrogels through future research is crucial.

**Figure 7:**
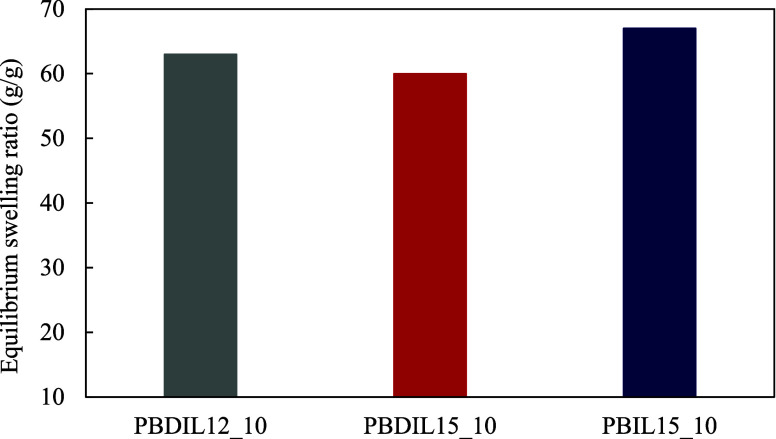
Water absorption capacities of the hydrogels.

**Figure 8:**
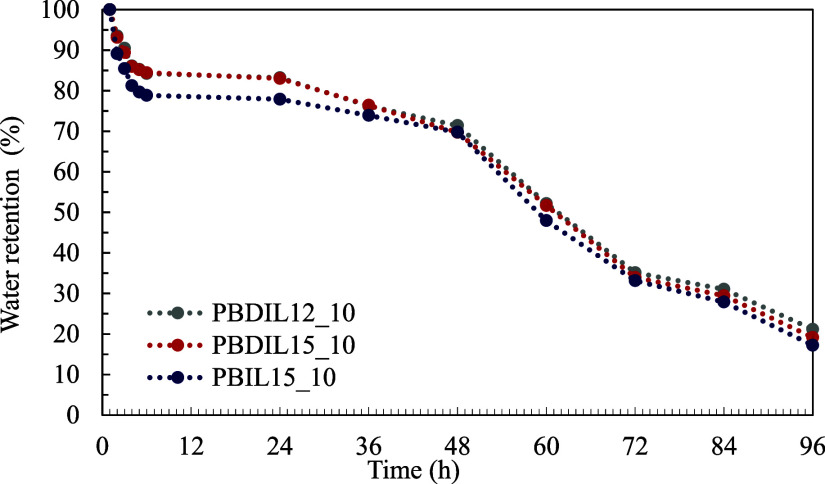
Water retention ratio of hydrogels under under atmospheric conditions.

### 3.5 ATR-FTIR Analysis of Treated Cotton Fabrics

After the successful preparation of the triazolium-containing hydrogel, we applied this robust material to the surface of cotton fabrics to enhance their flame retardancy and antibacterial activity. The interactions and structural formations among PVA, borax and triazolium salts within the hydrogels are confirmed using ATR-FTIR spectra, as presented in Figure 9. In the PB hydrogel spectrum, the C–H alkyl stretching vibration peaks of PVA was observed at 2910 cm^–1^, whereas the C–O stretching vibration peak of the secondary alcohol appeared at 1095 cm^–1.^ ^44^ Furthermore, the PB hydrogel spectrum exhibited characteristic peaks of borax and borate. For example, the peaks at 1403 and 1340 cm^–1^ are attributed to the asymmetric stretching of B–O–C. Specifically, the peak at 1403 cm^–1^ corresponds to the tetrahedral complex, whereas the peak at 1340 cm^–1^ indicates the formation of a triangular complex. Additionally, the B–O stretching vibration peak at 830 cm^–1^ corresponds to the residual borate ion B­(OH)_4_^–^, and the small peak at 644 cm^–1^ is associated with the bending of the B–O–B bonds within the borate network.^45^ The presence of these peaks confirms the cross-linking between borax and the PVA chain, resulting in the formation of boronic ester bonds, and indicates the presence of a small amount of the residual borax salt B­(OH)_4_^–^ within the hydrogel. In the IR spectra of PBDIL12_100, PBDIL15_100 and PBIL15_100, the characteristic peaks at 1403, 830, and 644 cm^–1^ were retained, indicating that the incorporation of triazolium ionic salts did not disrupt the formation of the borate ester bond between borax and PVA. Furthermore, the C–H bending vibration peaks detected between 1000 and 1200 cm–^1^ provided additional evidence of interactions between the ionic salts and polymer structure.^41^ The ATR-FTIR spectra indicate the presence of dynamic borate bonds as well as hydrogen bonds within and between the components, suggesting the successful formation of a three-dimensional network cross-linking structure in the triazolium-containing hydrogels. Overall, the FTIR analysis results confirm the successful fabrication of the targeted triazolium-containing hydrogel-treated cotton fabrics.

**Figure 9:**
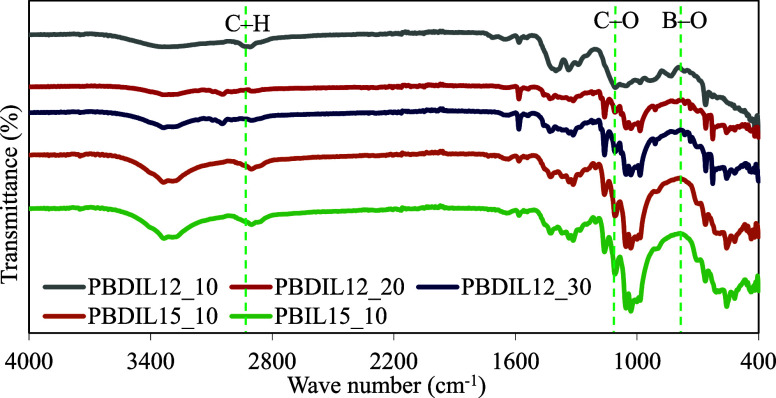
ATR/FT-IR spectrum of the hydrogel-treated cotton fabric.

### 3.6 SEM Morphology and EDS Analysis of Treated Cotton Fabrics

SEM analysis was performed on PBDIL12_10, PBDIL15_10, and PBIL15_10 to examine their surface morphology. As shown in the SEM images in Figure 10, significant deposition was observed on the fiber surfaces. This indicates that, due to the treatment of cotton fabric at different doses, the triazolium salt-containing hydrogel was successfully fixed onto the fabric surface using the pad-dry-cure method. The SEM images confirmed that the fabrics were effectively treated with flame retardants. To verify the incorporation of triazolium salt into the hydrogel structure, the elemental composition of the cotton fabric surface was analyzed using EDS. As shown in Figure 11, the primary elements detected on the treated fabric surface were O, C, N, and Br. The thermal stability of triazolium salt-containing flame retardant hydrogel based on nitrogen was studied with thermogravimetric analysis. The degradation activation energies, decomposition temperatures and weight loss rates were altered by the flame retardant hydrogels. Incorporation of DIL15 and IL15 can lower the activation energies at early stages of the degradation. The incorporation of flame-retardant components decreased the activation energy at lower degradation levels but increased it at higher degradation levels.^46^

**Figure 10:**
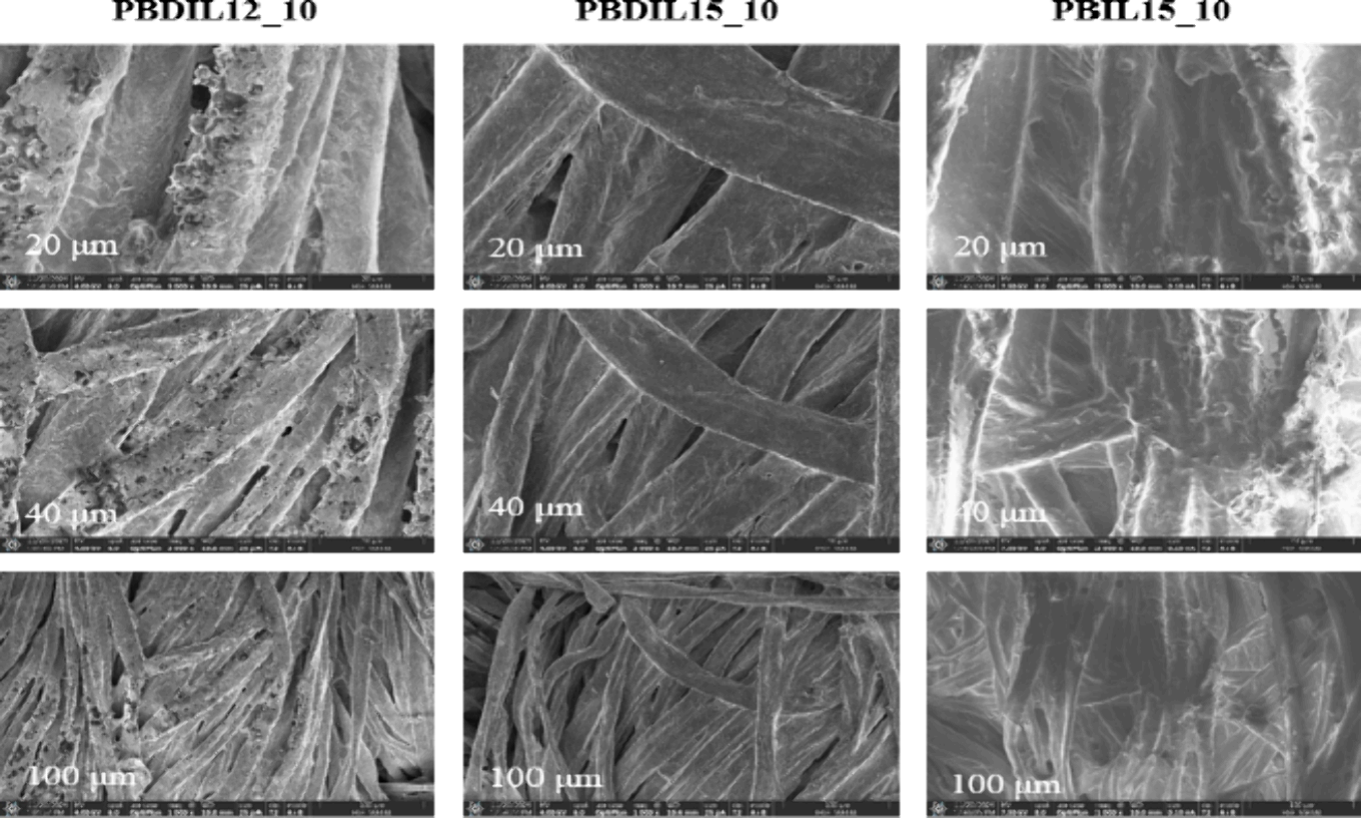
Scanning electron microscopy images of treated cotton fabrics.

**Figure 11:**
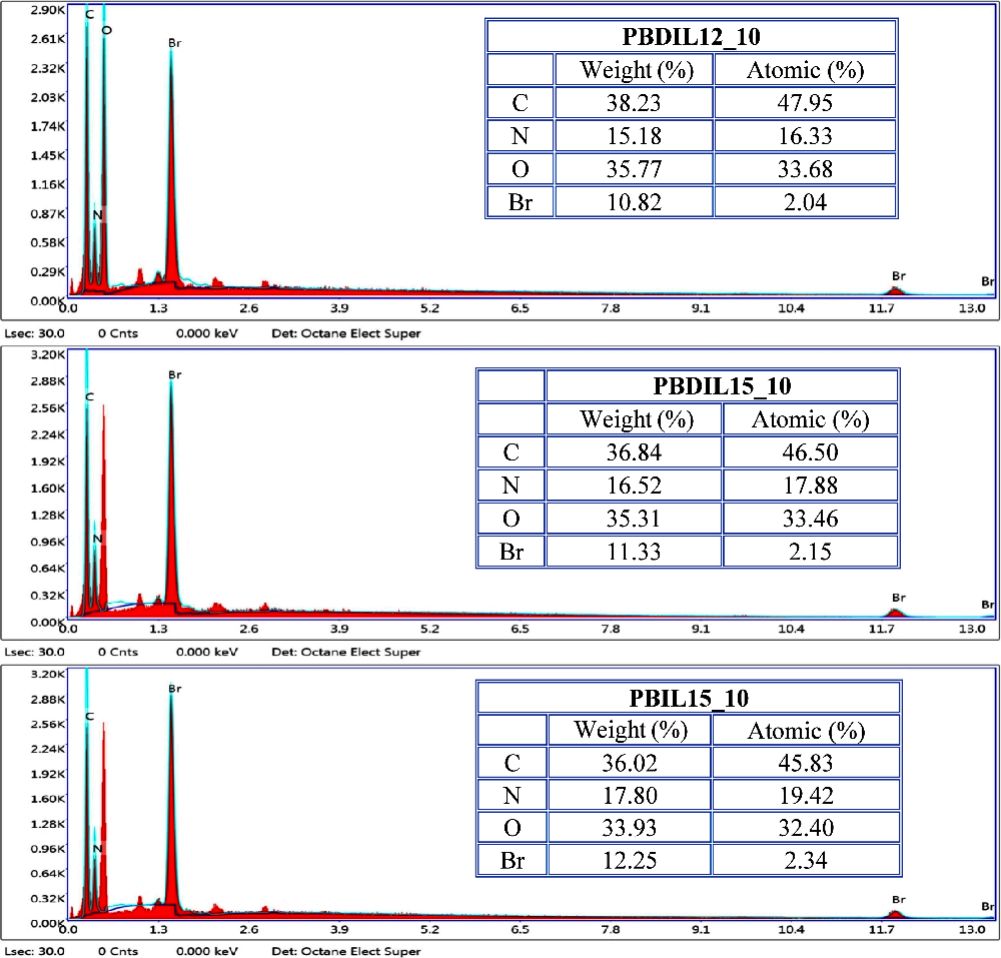
EDS spectral analysis of the surfaces of treated cotton fabrics.

### 3.7 Effects of Flame Retardancy on Cotton Fabrics

Vertical flammability and LOI tests were conducted to assess the flame retardancy of cotton fabrics treated with different concentrations of PBIL15_10, PBDIL15_10, PBDIL12_10, PBDIL12_20 and PBDIL12_30. The images and data obtained via these tests are presented in Figure 12 and Table 6. The addition of IL15 had no noticeable effects on flame retardancy. In contrast, PBDIL12_20 demonstrated a substantial flame-retardant effect, exhibiting a mass increase of 46.60% and an LOI of 37.2%. Meanwhile, the LOI and weight gain of PBDIL12_30 were 31.8 and 38.60%, respectively.

**Table 6:** Flame-Retardant Performance of the Samples

	concentration of Flame Retardants (wt %)							
samples	PVA	borax	IL15	DIL15	DIL12	mass increase (%)	damaged length (mm)	LOI (%)
control						0	burned	
PBIL15_10			10			5.07 ± 0.53	burned	
PBDIL15_10				10		8.31 ± 0.55	48	
PBDIL12_10	1.5	2			10	12.15 ± 1.92	45	22.8
PBDIL12_20					20	46.60 ± 3.34	38	37.2
PBDIL15_30					30	38.60 ± 1.68	41	31.8

**Figure 12:**
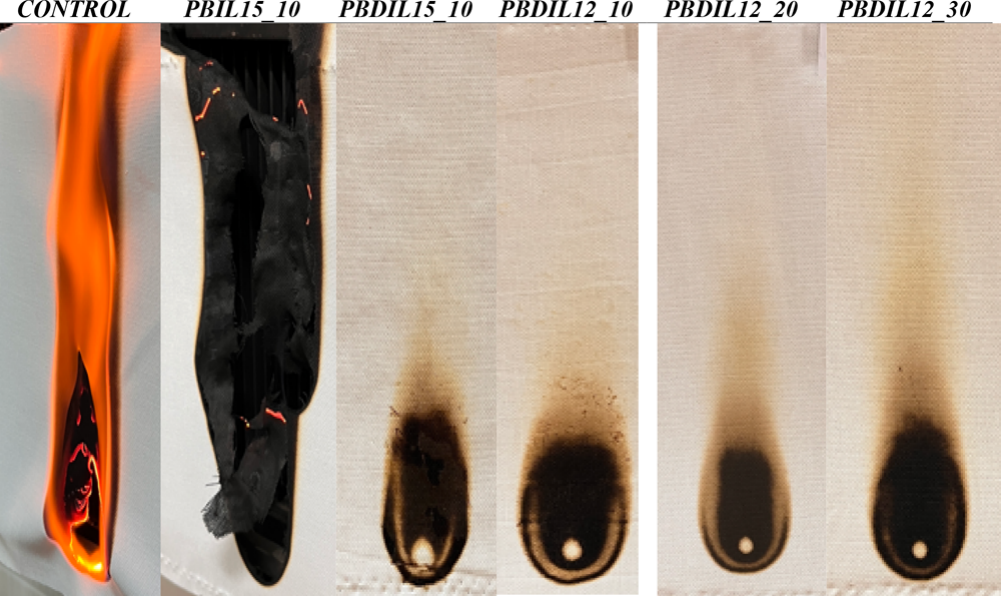
Flame test photos of the samples.

### 3.8 TG-FTIR Analysis on Cotton Fabrics

The composition of gaseous products during thermal decomposition was analyzed by TGA-FTIR under an air atmosphere, and shown in Figure 13 and SI (Figure S13), Six wavebands were identified at 3750–3500, 3020–2790, 2400–2260, 1840–1650, 1560–1339, and 1232–1030 cm^–1^, corresponding to the release of H_2_O, hydrocarbons, CO_2_, CO, carbonyl compounds, and ether compounds, respectively.^47^ Figure 13 indicates that all three samples released the aforementioned gas-phase substances during the test, but the release time and amount were significantly different. For these gaseous products, CO_2_ and water release occurs due to dehydration processes or the decomposition of organic compounds from the treated cotton fabric during pyrolysis, while carbonyl, C–O–C, and–CH_3_/–CH_2_ groups resulted from the depolymerization process.^6^ Accordingly, cotton fabrics treated with PBDIL12_10 and PBDIL15_10 showed higher absorption intensities of CO_2_ and H_2_O compared to PBIL15_10, which diluted flammable gases and oxygen during combustion. Moreover, the cotton fabric treated with PBDIL12_10 exhibited a significantly more thermally stable compound, with a peak intensity of volatile combustible gases (ethers, carbonyls, CO) and fewer easily decomposable groups. The reduction of the flammable volatiles resulted in less “fuel″ entering the flame zone. In conclusion, the incorporation of PBDIL12_10 into treated cotton fabric led to a decrease in the release of flammable volatiles in the gaseous phase during combustion.

**Figure 13:**
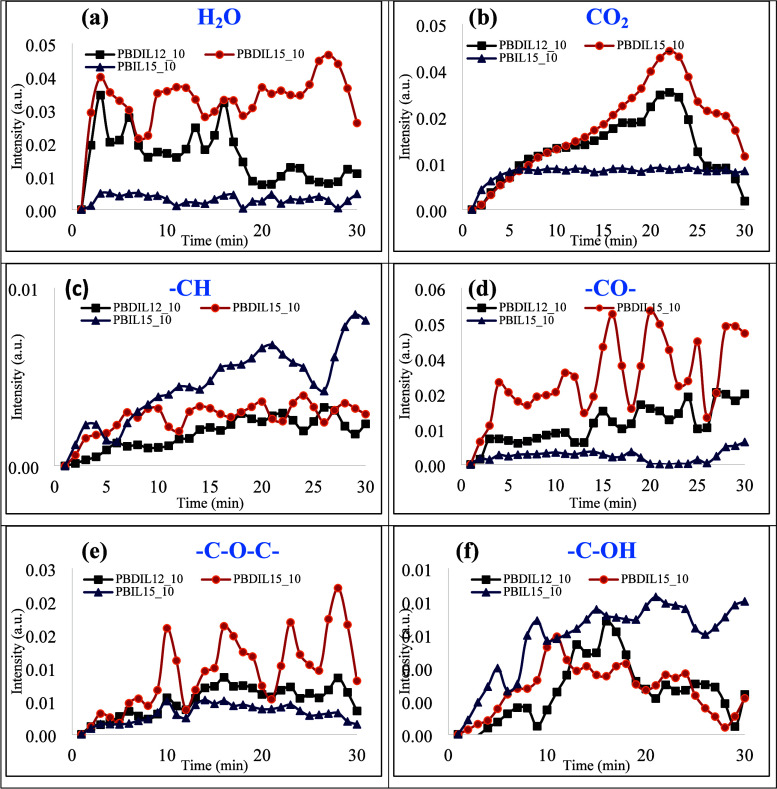
Intensities of the characteristic peaks of the pyrolysis products of treated cotton (a) H_2_O; (b) CO_2_; (c) hydrocarbon (−CH); (d) CO; (e) carbonyl (−C–O–C−); and (f) ether (−C–OH).

### 3.9 Antibacterial Properties of the Treated Cotton Fabrics

The antibacterial properties of the treated fabrics were assessed using E. coli and S. aureus, which are typical Gram-negative and Gram-positive bacteria, respectively. Table 7 indicates that the treatment of the fabrics using the triazolium-containing hydrogel was effective, as evidenced by the high efficacy of the treated fabrics against both Gram-positive (S. aureus) and Gram-negative (*E. coli*) bacteria. However, the antibacterial activity of the hydrogel without triazolium was more effective against Gram-negative bacteria (E. coli) than against Gram-positive bacteria (S. aureus).

**Table 7:** Antibacterial Properties of the Treated Fabrics against S. aureus and E. coli

	reduction (%)	
samples	S. aureus	E. coli
untreated cotton	18.000	13.950
PB	84.941	>99.994
PBIL15_10	>99.997	>99.996
PBDIL15_10	>99.997	>99.996
PBDIL12_10	>99.998	>99.998
PBDIL12_20	>99.998	>99.998
PBDIL12_30	>99.998	>99.998

### 3.10 Effects on the Strength of Cotton Fabrics

Tensile strength is a crucial physical parameter for evaluating and controlling the performance of cotton fabrics after treatment, particularly in applications such as fire and rescue operations.^48^ Figure 14 illustrates the tensile strength of the pretreated and hydrogel-treated cotton fabrics. The tensile strength of the pretreated cotton fabric was lower than that of the hydrogel-treated cotton fabric. The higher tensile strength of the hydrogel-treated cotton fabrics is primarily because of the diffusion of a certain amount of water stored in the hydrogel into the fibers, which improves their stress concentration. Clearly, the introduction of the hydrogel onto the surface of the cotton fabrics has contributed to increase their mechanical strength. Furthermore, the tensile strength of the hydrogel-treated cotton fabrics consistently increased with rising triazolium ionic salt concentration in the hydrogels. In general, the strength of the cotton fabric samples increased with increasing concentration of the dicationic triazolium ionic salt up to 20 wt %. However, the PBDIL12_30 sample, with a reinforcement of >20 wt % (30 wt %), may exhibit lower mechanical strength, possibly because of the lower weight gain of the cotton fabrics.

**Figure 14:**
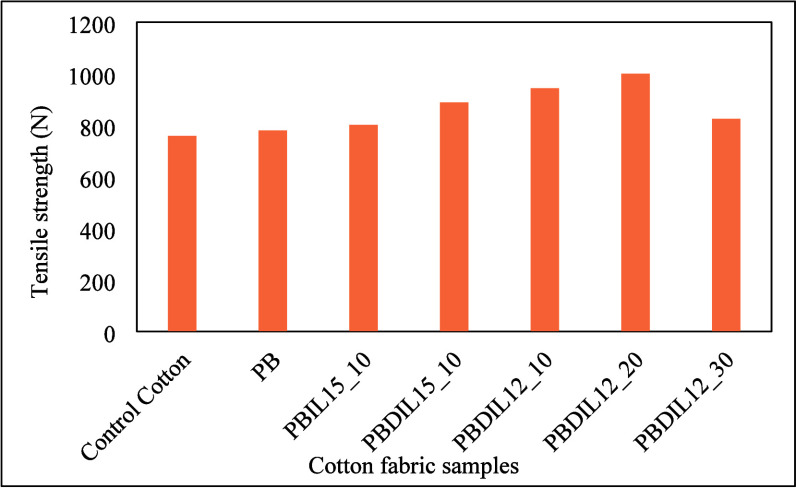
Tensile strength of cotton fabrics.

### 3.11 Effects of Triazolium Salts on Cell Viability in HaCaT and BJ Cell Lines

The effects of the synthesized triazolium salts (IL15, DIL12, DIL15) on cell viability were evaluated in human keratinocyte (HaCaT) and dermal fibroblast (BJ) cell lines following 48 h of treatment using the MTT assay. Cells were exposed to five concentrations (1, 10, 100, 250, and 500 μM), and viability was expressed as a percentage relative to untreated controls (Figure 15). In HaCaT cells, exposure to 1 and 10 μM concentrations did not significantly affect cell viability, with values remaining above 90%. However, a concentration-dependent reduction became evident at higher doses. Treatment with 100 μM resulted in a moderate decrease (∼75% viability), while 250 and 500 μM led to more pronounced reductions, with cell viability decreasing to approximately 55 and 35%, respectively (*p* < 0.05 compared to control).

**Figure 15:**
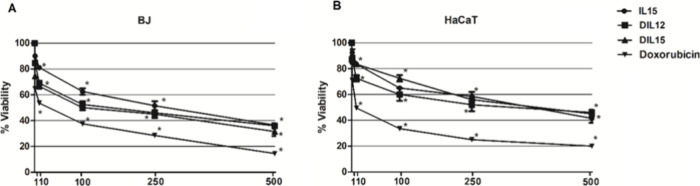
MTT assay results showing the effect of triazolium salts on cell viability after 48 h. (A) BJ fibroblasts; (B) HaCaT keratinocytes. Cells were treated with 1, 10, 100, 250, and 500 μM concentrations of triazolium salts. Data represent mean ± SD from three independent experiments (*n* = 3).

In BJ fibroblasts, a similar concentration-dependent pattern was observed, although the cells demonstrated slightly greater resistance. At 1 and 10 μM, viability remained above 95%, and a mild decrease (∼85%) was detected at 100 μM. At 250 μM, cell viability dropped to around 65%, and at the highest concentration (500 μM), viability was reduced to nearly 45% (*p* < 0.05).

These findings indicate that while low concentrations of triazolium salts have minimal impact on normal human cells, higher doses-particularly 250 and 500 μM-lead to significant reductions in viability after 48 h of treatment. BJ fibroblasts exhibited slightly greater tolerance than HaCaT cells, demonstrating distinct cellular responses to the compounds.

## 4 Conclusions

In this study, we prepared a novel environmentally friendly flame-retardant and antibacterial triazolium-containing hydrogel and applied it to cotton fabrics. The synthesized triazolium salts were incorporated in different concentrations into the hydrogel to evaluate their flame retardancy and antibacterial effects due to the nitrogen-rich salts based on the combination of 1-methyl-1,2,4-triazole. K–A–S, F–W–O and Starink methodologies were used to determine the thermal degradation kinetic parameters. From the experimental data, the average activation energies for thermal degradation were determined as 103.506, 192.978, and 246.482 kJ/mol for IL15, DIL15 and DIL12, respectively. These results suggest that a short alkyl chain and the addition of a triazolium ring enhance the thermal stability of triazolium-based flame retardants, primarily because of the deceleration of thermal degradation due to carbonization. The flame retardancy performance of the hydrogel-treated cotton fabrics was assessed using vertical flammability tests. The treated fabrics exhibited effective flame-retardant properties due to promote generating more noncombustible volatiles in the gaseous phase, achieving a maximum LOI of 37.2%. The antibacterial activity of the treated fabrics was evaluated against *E. coli* and S. aureus, and the bacteriostatic rates of the synthesized materials exceeded 99% against both the bacteria. Our findings provide valuable insights into the relation between the triazolium content in hydrogels and their flame-retardant and antibacterial properties. These results offer crucial information for the design and manufacture of effective flame-retardant and antibacterial protective clothing for safeguarding individuals from fire hazards and health risks. Additionally, the treated fabrics had little effect on tensile strength and whiteness, which will be further improved with different hydrogel treatment parameters in our future research.

## Supplementary Material



## Notes

Chemicals used in the study; ^1^H NMR spectra of 1-methyl-1,2,4 triazole; ^13^C NMR spectra of 1-methyl-1,2,4 triazole; ^1^H NMR spectra of 1,2-bis­(1-methyl-1H-1,2,4 triazol-4-ium) ethane dibromide (DIL12); ^13^C NMR spectra of 1,2-bis­(1-methyl-1H-1,2,4 triazol-4-ium) ethane dibromide (DIL12); ^1^H NMR spectra of 1,5-bis­(1-methyl-1H-1,2,4 triazol-4-ium) pentane dibromide (DIL15); ^13^C NMR spectra of 1,5-bis­(1-methyl-1H-1,2,4 triazol-4-ium) pentane dibromide (DIL15); ^1^H NMR spectra of 1-methyl-4-pentyl-1H-1,2,4-triazol-4-ium bromide (IL15); ^13^C NMR spectra of 1-methyl-4-pentyl-1H-1,2,4-triazol-4-ium bromide (IL15); calculations of activation energy for DIL12 using the KAS, OFW, and STARINK methods; calculations of activation energy for DIL15 using the KAS, OFW, and STARINK methods; calculations of activation energy for IL15 using the KAS, OFW, and STARINK methods; TG graphs of PBIL15_10, PBDIL15_10, PBDIL12_10 at a 20 °C/mins heating rate; and TG-FTIR spectra of pyrolysis products of PBDIL12_10, PBDIL15_10, and PBIL15_10 (PDF)
