# Supplementary material for: Application of Novel Triazolium-Containing Hydrogels to Cotton Fabrics: Evaluation of Their Flame Retardancy and Antibacterial Properties
Source: ACS Omega. 2025 May 23;10(21):21486–98. doi: 10.1021/acsomega.5c00281 (PMC12138650; doi:10.1021/acsomega.5c00281)
Supplement: Supplementary file 1 [file ao5c00281_si_001.pdf]

Supplementary Information

Application of novel triazolium-containing hydrogels to cotton fabrics: Evaluation of their flame retardancy and antibacterial properties

Nejmi Söyler[1]; Eylen Sema Dalbaşı[2]; Süleyman Ilhan[3]; Hayati Türkmen[4,*]

[1] Graduate School of Natural and Applied Sciences, Materials Science and Engineering, Ege University, 35100 Bornova, Izmir, Turkey

[2] Department of Textile Engineering, Ege University, 35100 Bornova, Izmir, Turkey

[3]Department of Biology, Faculty of Engineering and Natural Sciences, Manisa Celal Bayar University, Manisa, Türkiye

[4] Department of Chemistry, Faculty of Science, Ege University, 35100 Bornova, Izmir, Turkey

*** Corresponding author e-mail:** hayati.turkmen@ege.edu.tr

Table S1. Chemicals used in the study 2

Figure S1. ^{1}H-NMR spectrum of 1-methyl-1,2,4 triazole. 3
Figure S2. ^{13}C-NMR spectrum of 1-methyl-1,2,4 triazole. 3
Figure S3. ^{1}H-NMR spectrum of 1,2-Bis(1-methyl-1H-1,2,4 triazol-4-ium) ethane dibromide (DIL12). 4
Figure S4. ^{13}C-NMR spectrum of 1,2-Bis(1-methyl-1H-1,2,4 triazol-4-ium) ethane dibromide (DIL12). 4
Figure S5. ^{1}H-NMR spectrum of 1,5-Bis(1-methyl-1H-1,2,4 triazol-4-ium) pentane dibromide (DIL15). 5
Figure S6. ^{13}C-NMR spectrum of 1,5-Bis(1-methyl-1H-1,2,4 triazol-4-ium) pentane dibromide (DIL15). 5
Figure S7. ^{1}H-NMR spectrum of 1-methyl-4-pentyl-1H-1,2,4-triazol-4-ium bromide (IL15). .6
Figure S8. ^{13}C-NMR spectrum of 1-methyl-4-pentyl-1H-1,2,4-triazol-4-ium bromide (IL15). 6
Figure S9. Calculation of activation energy for DIL12 using the KAS, OFW and STARINK methods. 7
Figure S10. Calculation of activation energy for DIL15 using the KAS, OFW and STARINK methods. 8
Figure S11. Calculation of activation energy for IL15 using the KAS, OFW and STARINK methods. 9
Figure S12. TG graphs of PBIL15_10, PBDIL15_10, PBDIL12_10 at 20 °C/min heating rate. 10

Figure S13. TG-FTIR spectra of pyrolysis products of (a) PBDIL12_10, (b) PBDIL15_10, and (c) PBIL15_10..11

Table S1. Chemicals used in the study.

Chemical Name	Molecular Structure	Molecular Formula	CAS Number	Purity (%)
**1H-1,2,4-Triazole**	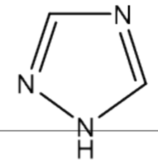	$C_2H_3N_3$	288-88-0	99
**1,2-Dibromoethane**	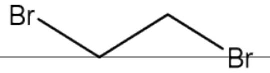	$C_2H_4Br_2$	106-93-4	98
**1,5-Dibromopentane**	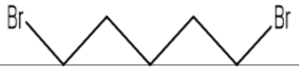	$C_5H_{10}Br_2$	111-24-0	98
**1-Bromopentane**	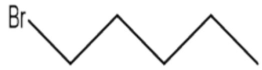	$C_5H_{11}Br$	110-53-2	98
**Polyvinyl alcohol**	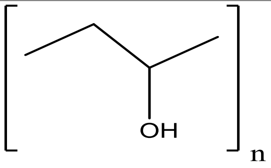	$[CH_2CH(OH)]_n$	9002-89-5	95
**Borax (Sodium tetraborate)**	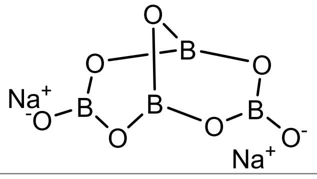	$Na_2B_4O_7$	1333-73-9	99

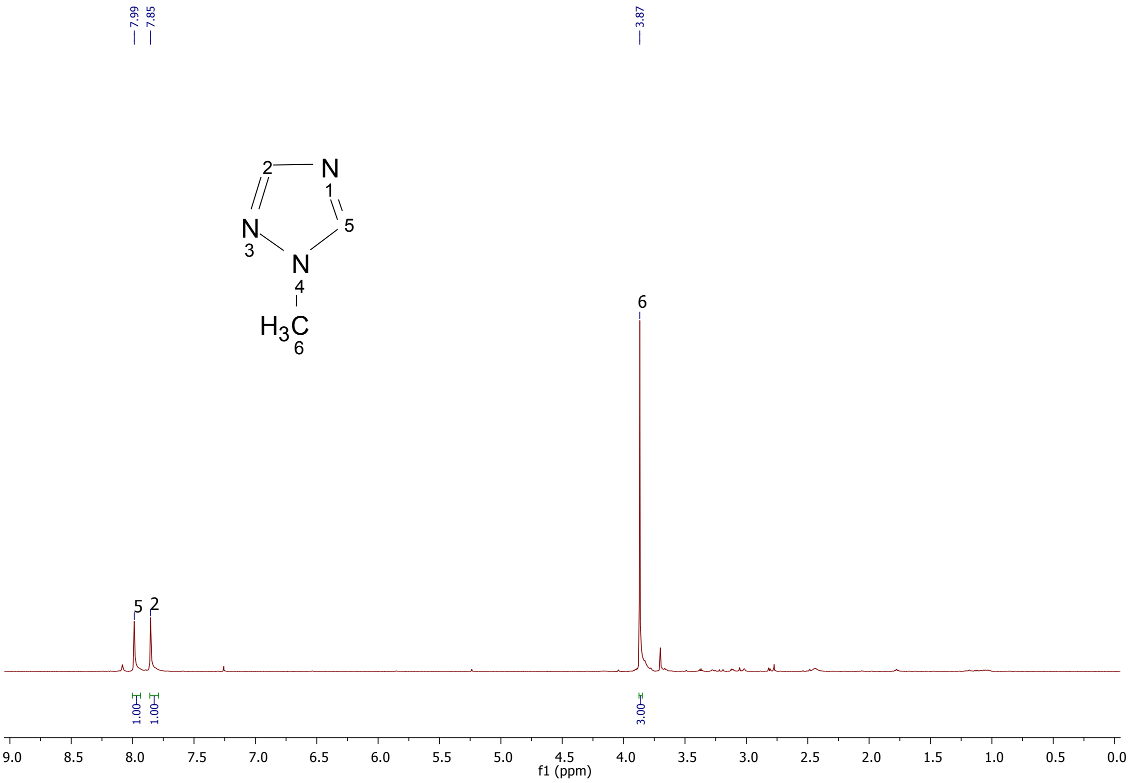

Figure S1. ^{1}H-NMR spectrum of 1-methyl-1,2,4 triazole.

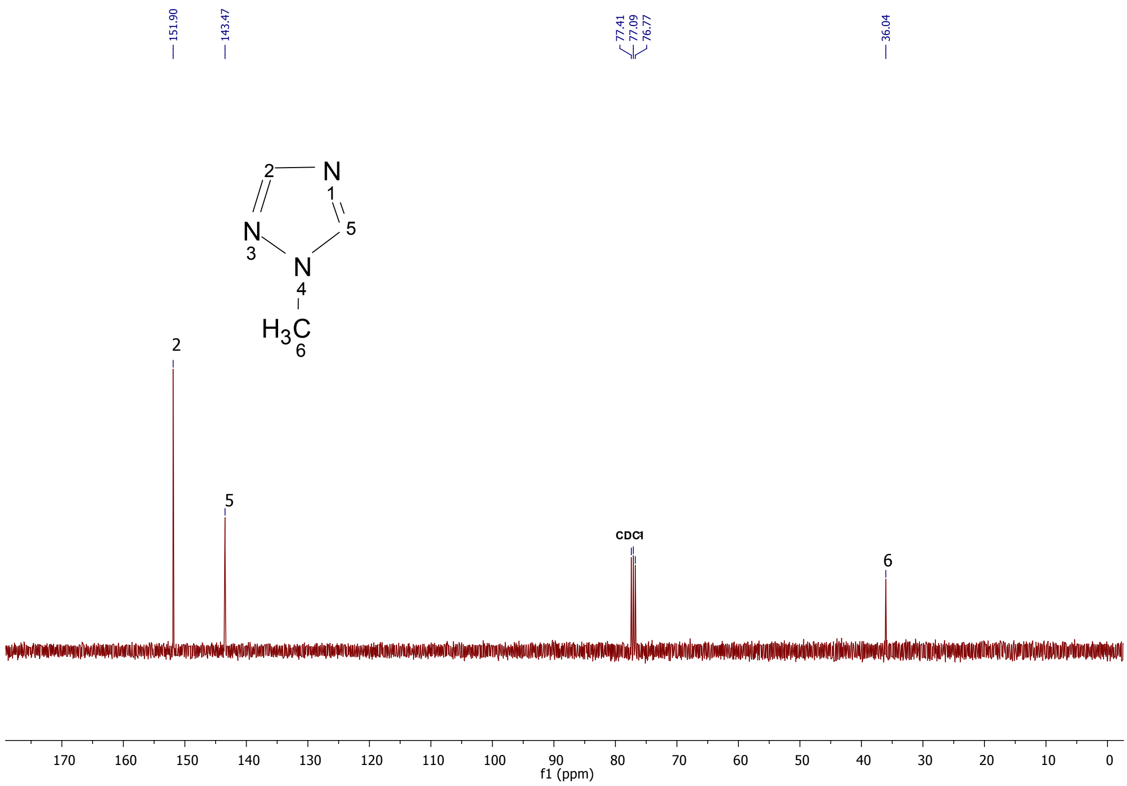

Figure S2. ^{13}C-NMR spectrum of 1-methyl-1,2,4 triazole.

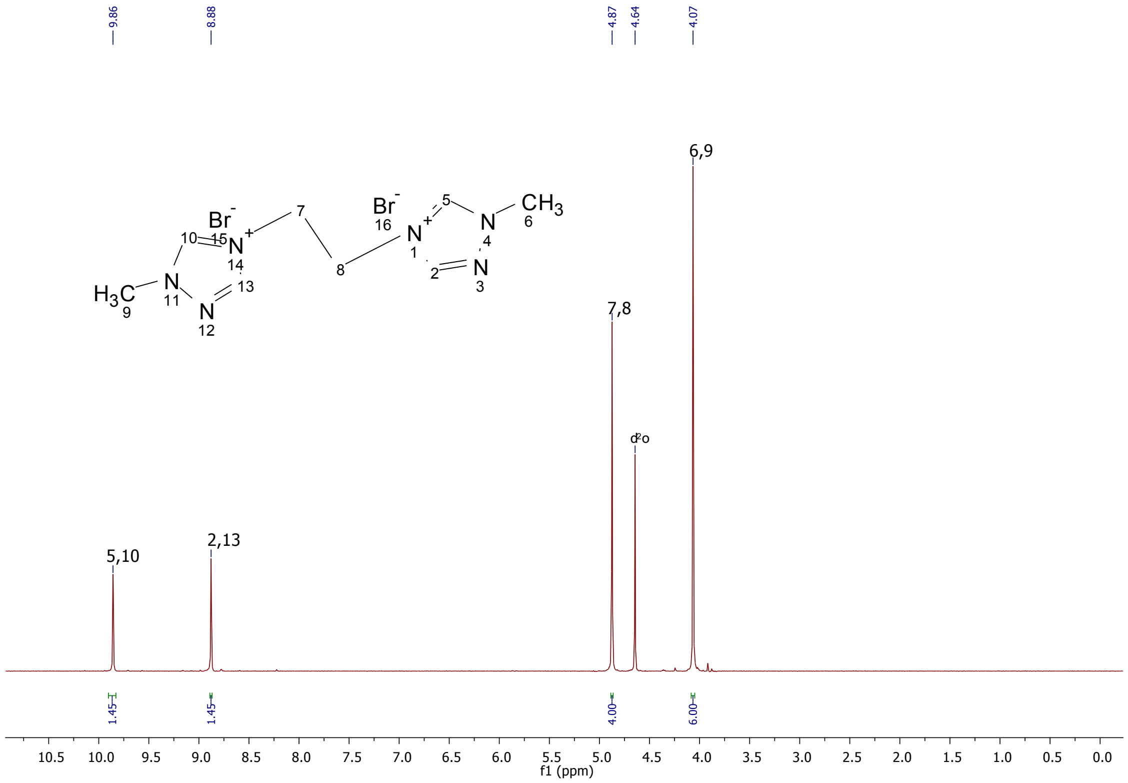

Figure S3. ^{1}H-NMR spectrum of 1,2-Bis(1-methyl-1H-1,2,4 triazol-4-ium) ethane dibromide (DIL12).

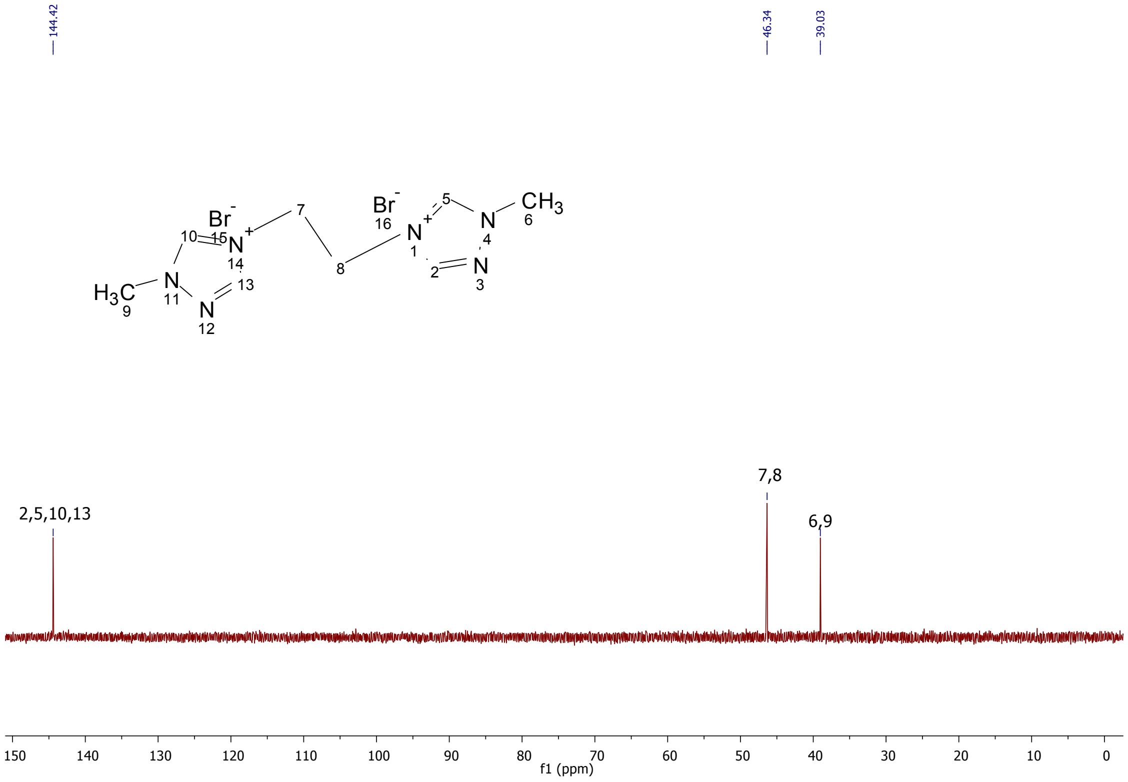

Figure S4. ^{13}C-NMR spectrum of 1,2-Bis(1-methyl-1H-1,2,4 triazol-4-ium) ethane dibromide (DIL12).

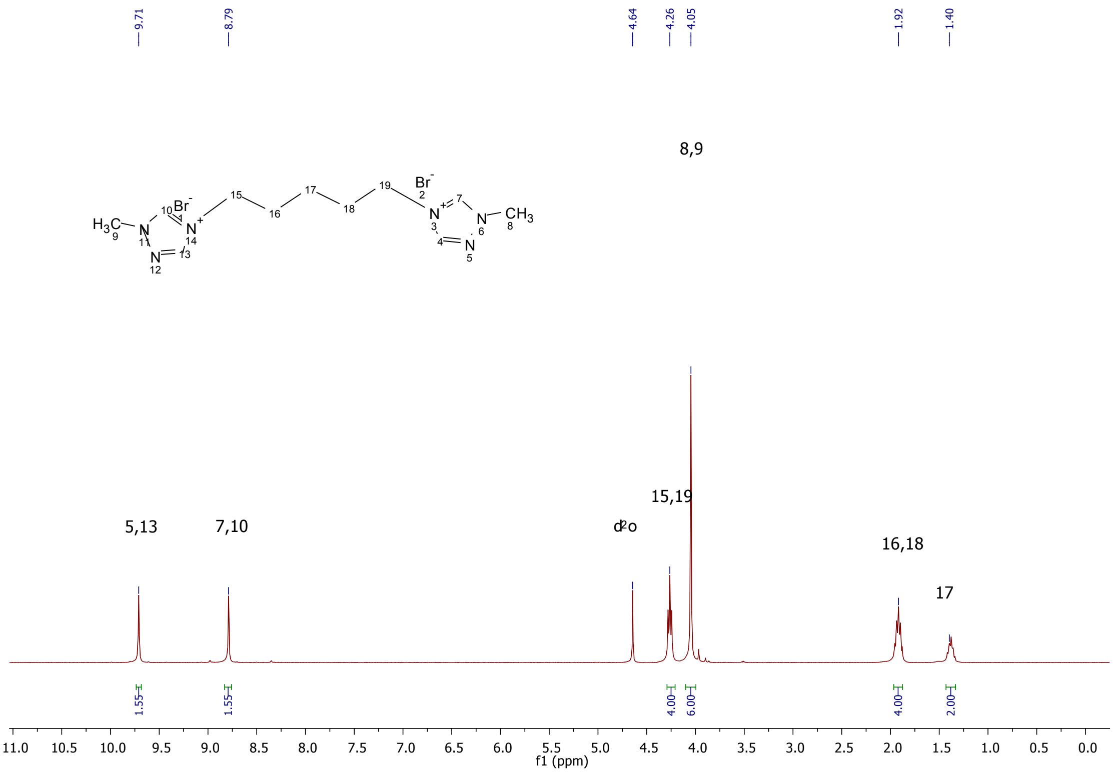

Figure S5. ^{1}H-NMR spectrum of 1,5-Bis(1-methyl-1H-1,2,4 triazol-4-ium) pentane dibromide (DIL15).

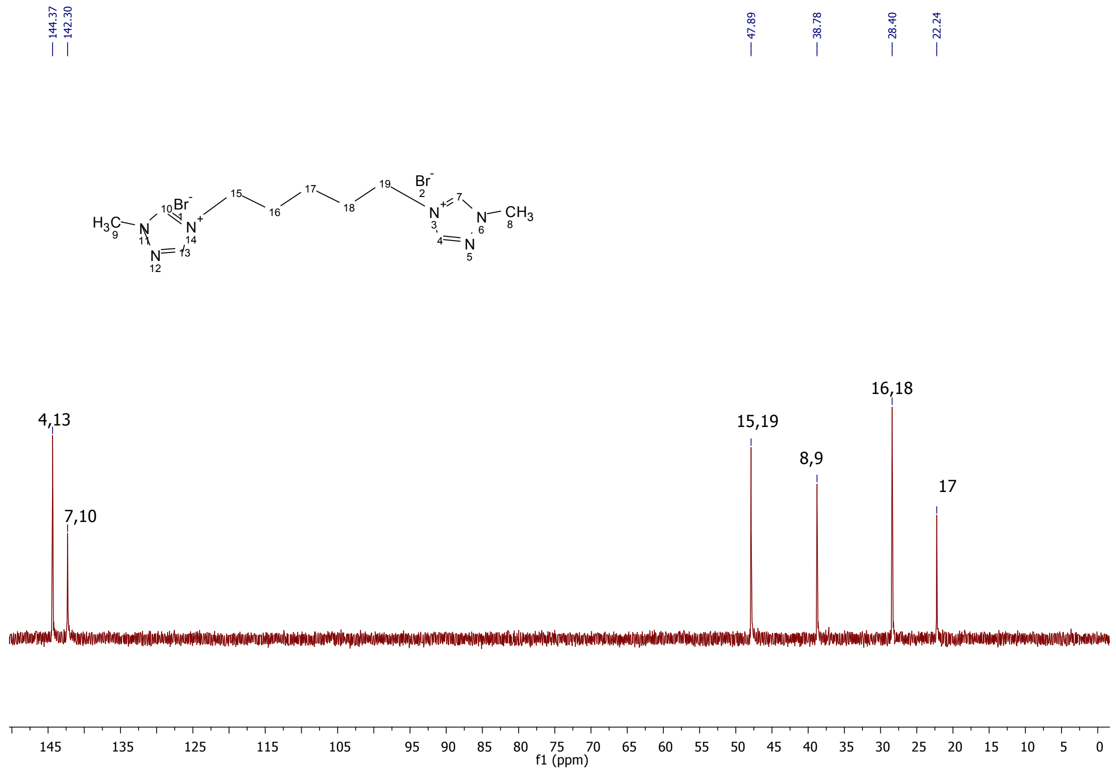

Figure S6. ^{13}C-NMR spectrum of 1,5-Bis(1-methyl-1H-1,2,4 triazol-4-ium) pentane dibromide (DIL15).

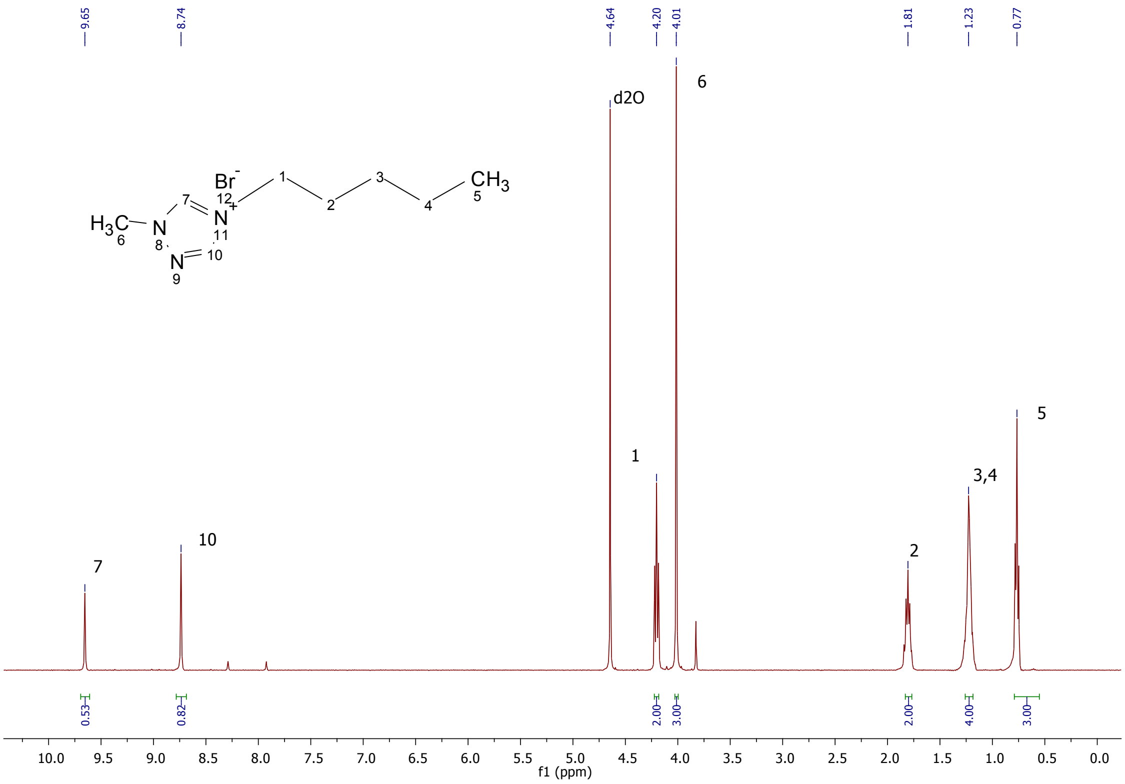

Figure S7. ^{1}H-NMR spectrum of 1-methyl-4-pentyl-1H-1,2,4-triazol-4-ium bromide (IL15).

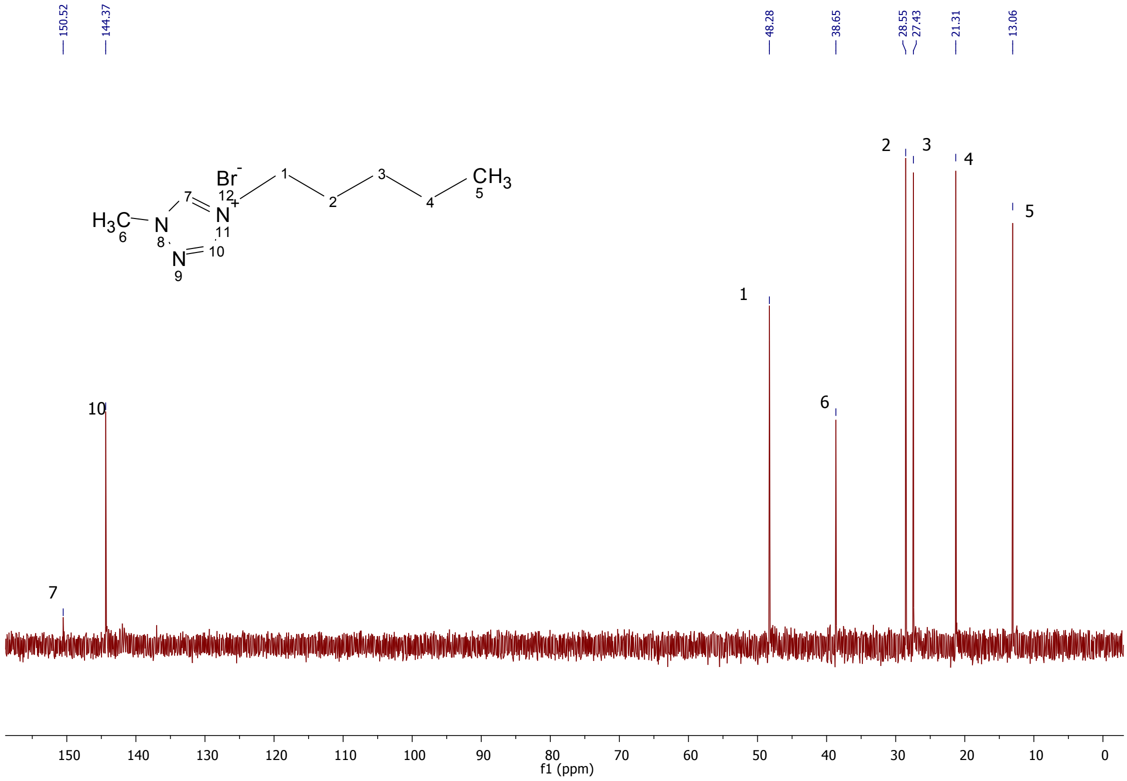

Figure S8. ^{13}C-NMR spectrum of 1-methyl-4-pentyl-1H-1,2,4-triazol-4-ium bromide (IL15).

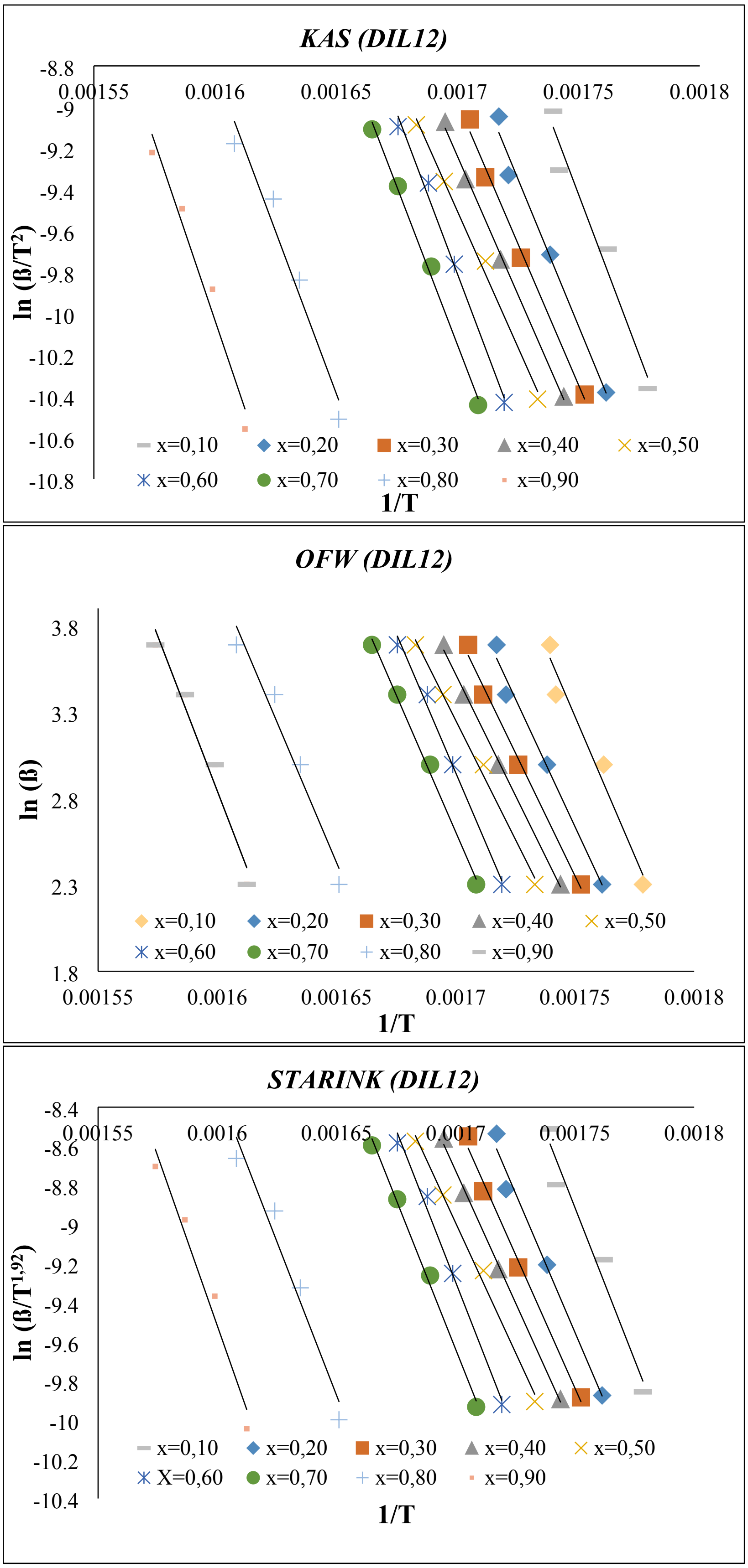

Figure S9. Calculation of activation energy for DIL12 using the KAS, OFW and STARINK methods.

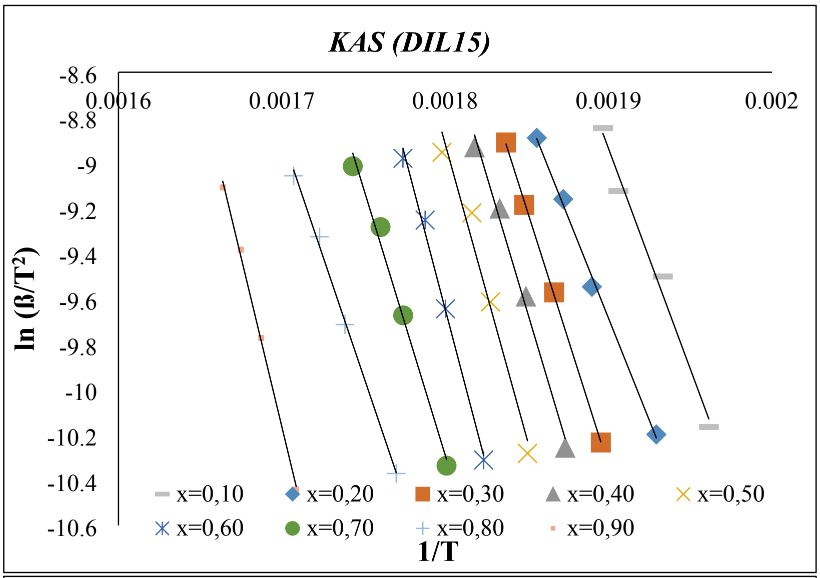

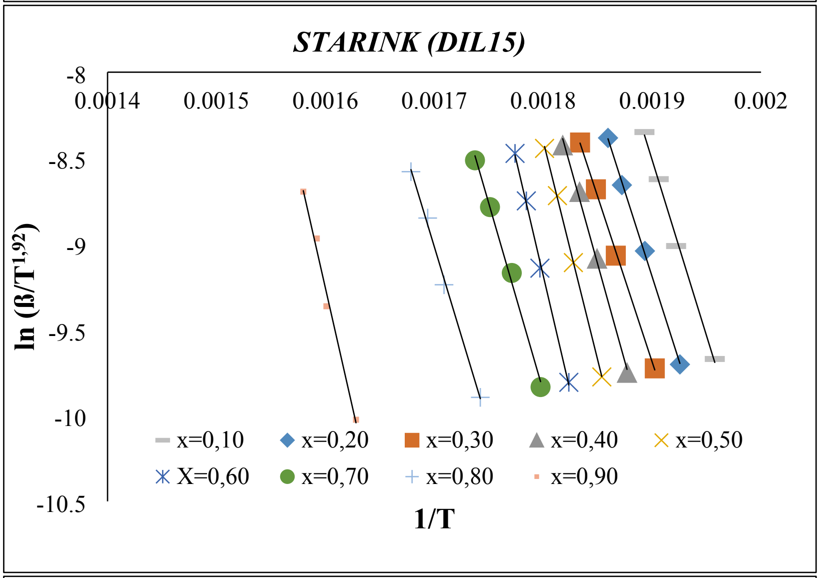

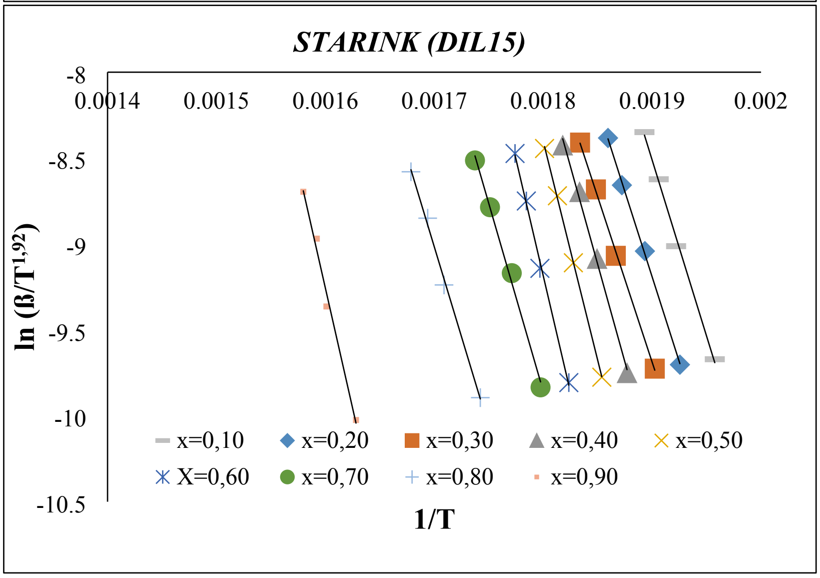

Figure S10. Calculation of activation energy for DIL15 using the KAS, OFW and STARINK methods.

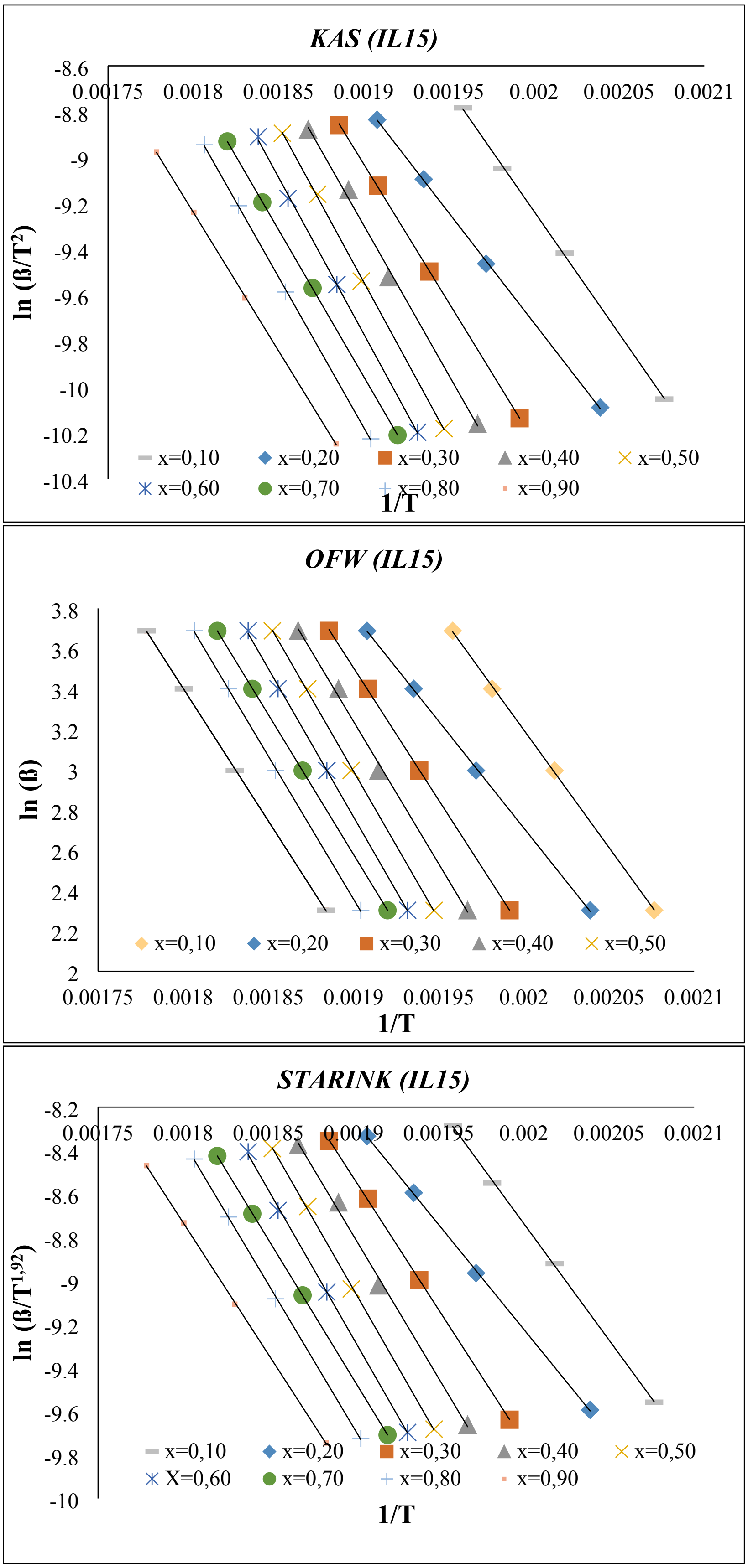

Figure S11. Calculation of activation energy for IL15 using the KAS, OFW and STARINK methods.

PBIL15_10

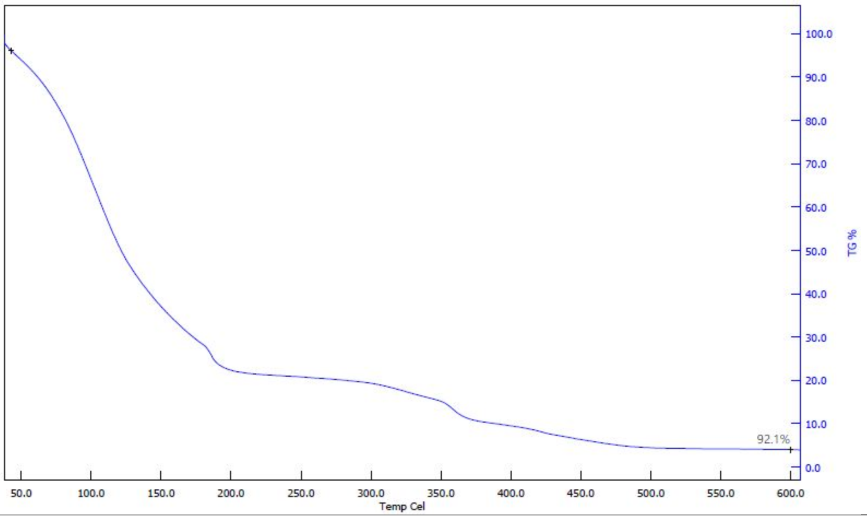

PBDIL15_10

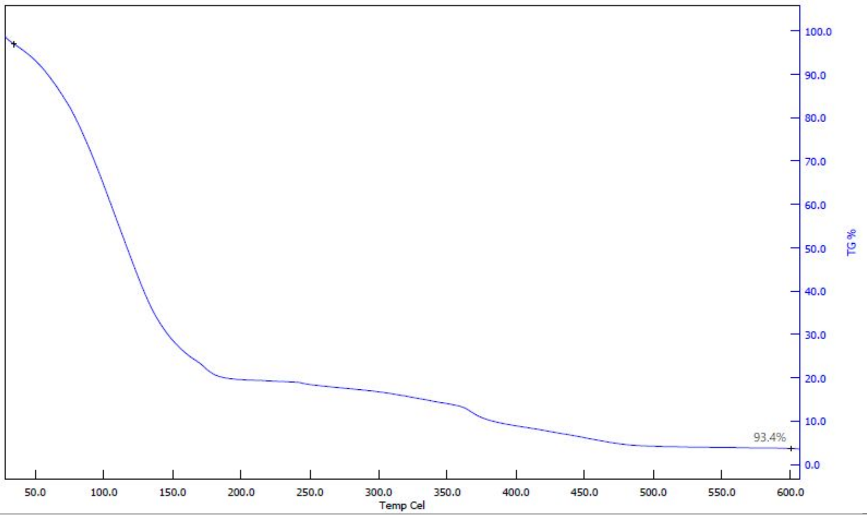

PBDIL12_10

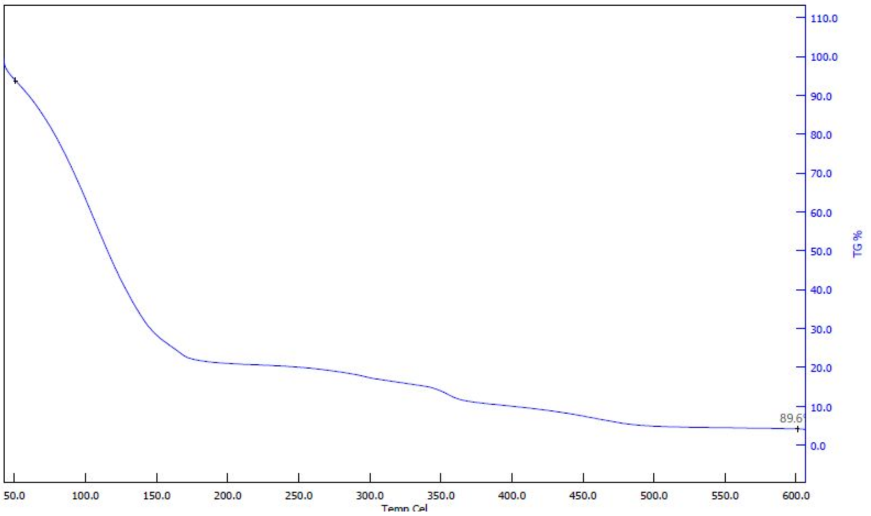

Figure S12. TG graphs of PBIL15_10, PBDIL15_10, PBDIL12_10 at 20 °C/min heating rate.

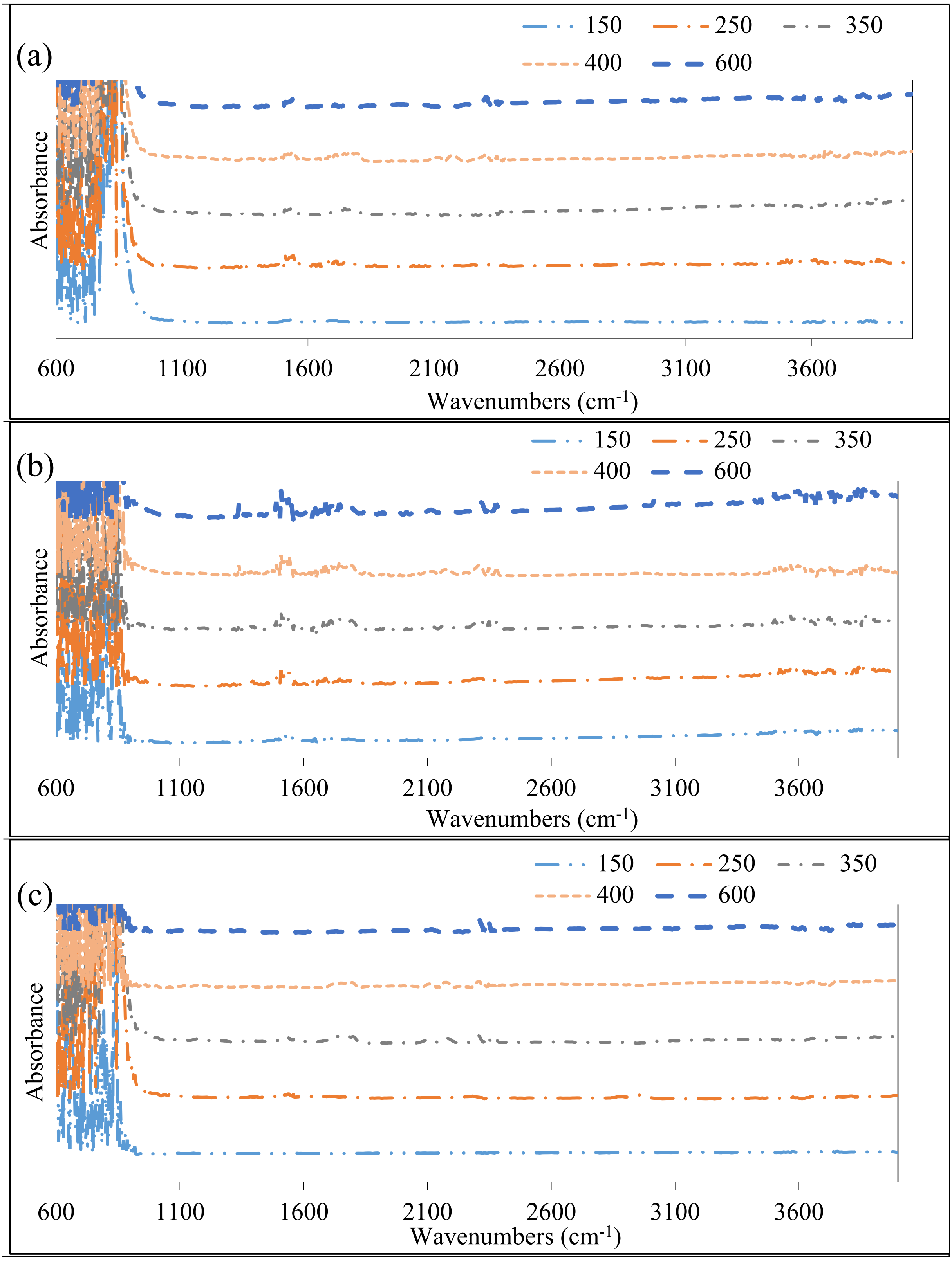

Figure S13. TG-FTIR spectra of pyrolysis products of (a) PBDIL12_10, (b) PBDIL15_10, and (c) PBIL15_10
